# Identification of DksA as a novel pro-inflammatory mediator of *Pseudomonas aeruginosa* under conditions mimicking chronic cystic fibrosis lung infection

**DOI:** 10.1080/21505594.2026.2670050

**Published:** 2026-05-12

**Authors:** Merel Wauters, Laura Bollé, Gilles De Meester, Sara Van den Bossche, Lucia Grassi, Delphi Van Haver, Sara Dufour, Simon Devos, Francis Impens, Eva Van Braeckel, Anna K. H. Hirsch, Marvin Whiteley, Xavier Saelens, Aurélie Crabbé

**Affiliations:** a Laboratory of Pharmaceutical Microbiology, Ghent University, Ghent, Belgium; b Respiratory Infection and Defense Lab (RIDL), Department of Internal Medicine and Paediatrics, Faculty of Medicine and Health Sciences, Ghent University, Ghent, Belgium; c Department of Respiratory Medicine, Ghent University Hospital, Ghent, Belgium; d VIB Proteomics Core, VIB, Ghent, Belgium; e Center for Medical Biotechnology, VIB, Ghent, Belgium; f Department of Biomolecular Medicine, Ghent University, Ghent, Belgium; g Helmholtz Institute for Pharmaceutical Research Saarland (HIPS) – Helmholtz Centre for Infection Research (HZI), Saarbrücken, Germany; h Department of Pharmacy, Saarland University, Saarbrücken, Germany; i PharmaScienceHub, Saarbrücken, Germany; j School of Biological Sciences and Center for Microbial Dynamics and Infection, Georgia Institute of Technology, Atlanta, Georgia, USA; k Department of Biochemistry and Microbiology, Ghent University, Ghent, Belgium

**Keywords:** *Pseudomonas aeruginosa*, virulence factors, inflammation, cystic fibrosis, proteomics, transcriptomics

## Abstract

Chronic infection with *Pseudomonas aeruginosa* is a major driver of airway inflammation, which plays a central role in the progression of cystic fibrosis (CF) lung disease. During long-term colonization, *P. aeruginosa* adapts to the CF lung by downregulating virulence factors and adopting a biofilm-associated, mucoid lifestyle. Despite the expected reduction in immune activation due to these adaptations, excessive inflammation persists, a paradox that remains poorly understood. Our objective was to identify novel bacterial mediators sustaining persistent inflammation by *P. aeruginosa* in the CF lung. To this end, we analyzed clinical *P. aeruginosa* CF isolates, cultured them in synthetic CF sputum medium, and exposed 3D lung epithelial cell cultures to the resulting cell-free supernatants. There was considerable variability in pro-inflammatory activity among the isolates, with a subset of the isolates inducing strong IL-8 secretion by the 3D cells despite low production of known virulence factors. Comparative proteomics analysis of the cell-free supernatants of pro-inflammatory and immunosuppressive isolates revealed several mediators not previously linked to inflammation. Thirteen of these candidate pro-inflammatory mediators were selected for further analysis. Using *P. aeruginosa* transposon mutants lacking the respective mediators, DksA (a transcription factor) was confirmed as an immunomodulatory mediator in the 3D lung model. Finally, analysis of existing transcriptomes of *P. aeruginosa* in CF sputum revealed that *dksA* was found to be one of the most strongly expressed genes in this patient population, highlighting the relevance of our findings. In conclusion, we identified a novel *P. aeruginosa* mediator that may contribute to CF airway inflammation.

## Introduction

Chronic infection by *Pseudomonas aeruginosa* is a major driver of adverse clinical outcomes in people with cystic fibrosis (pwCF) [1]. Although highly effective modulator therapy reduces the relative abundance of *P. aeruginosa* and leads to substantial clinical improvements, persistent infections continue to occur in many pwCF, underscoring the need for continued vigilance [2,3]. Long-term colonization of the CF lung arises from both intrinsic host defects, such as viscous mucus, impaired mucociliary clearance and dysregulated immunity, and the remarkable ability of *P. aeruginosa* to adapt and evade immune clearance [4,5]. The extensive genomic variability, metabolic adaptability, and phenotypic diversity of *P. aeruginosa* have contributed to its effectiveness as a dominant opportunistic pathogen in the CF airway [6–8].

During chronic infection, *P. aeruginosa* adapts to the CF lung by becoming less invasive and less virulent, enabling bacterial persistence while at the same time limiting extensive host damage [9]. To this end, *P. aeruginosa* downregulates several virulence factors, including proteolytic activity, type III secretion system (T3SS), and motility, and switches to a sessile biofilm-based and mucoid lifestyle that enhances tolerance to both host defense systems and antibiotics [4,9,10]. Additional adaptations include metabolic reprogramming to sustain growth under hypoxic mucus conditions and to use alternative nutrient sources, as well as selection of *P. aeruginosa* strains with mutations in quorum-sensing regulators such as *lasR*, and the emergence of hypermutator lineages and persister cells capable of long-term survival [4,10].

A paradox in chronic pseudomonal infection in pwCF is that, despite attenuated virulence, exacerbated airway inflammation persists – a phenomenon that remains poorly understood. Studies have demonstrated that late-stage *P. aeruginosa* CF isolates trigger robust cytokine release *in vitro, in vivo* using animal models, and in pwCF [11–14]. This excessive inflammatory response ultimately contributes to structural lung damage and a progressive decline in lung function [5,15]. While we recently highlighted the role of proteases (such as LasB) in immune evasion [16], the current study aims to identify previously unrecognized pro-inflammatory mediators involved in the excessive inflammation caused by *P. aeruginosa* during chronic infection in pwCF. To this end, *P. aeruginosa* was cultured under physiologically relevant conditions to stimulate the production of mediators likely expressed during *in vivo* infection. Specifically, we used synthetic CF sputum medium (SCFM2) to closely reproduce the nutritional environment of the CF lung and *P. aeruginosa* gene expression [17,18]. Furthermore, a three-dimensional (3D) alveolar epithelial cell model mimicking key phenotypic and functional characteristics of the native lung epithelium was used to assess host inflammatory responses [19–21]. A comparative proteomics analysis of a previously generated dataset was performed to characterize differences between pro-inflammatory and immunosuppressive CF isolates, while existing *P. aeruginosa* CF sputum transcriptomic datasets were leveraged to confirm *in vivo* expression of the identified mediators.

In this study, we advance the understanding of mediators driving persistent inflammation during chronic *P. aeruginosa* CF lung infection and highlight potential targets for the development of novel therapeutic interventions.

## Materials & methods

### Bacterial species, culture conditions, and supernatant preparation

*P. aeruginosa* isolates were previously obtained from the sputum samples of two individuals with CF (referred to as patient 1 and 6) [22]. Patient 1 had an early-stage infection (1 year), whereas patient 6 had a long-term chronic infection (>19 years). Throughout this paper, isolates are denoted as X/Y, where X is the isolate number and Y is the patient number. For example, 7/6 indicates isolate 7 from patient 6. In addition, several *P. aeruginosa* reference strains were included, namely, PAO1, AA44 (a late CF sputum isolate), and AMT 0023–30, a pediatric early CF isolate [23,24]. AMT 0023–30 was used as a positive control for quantification of pyocyanin production as it has been reported as a high producer, while PAO1 was used as a positive control for the quantification of pyoverdine [23,25]. For select experiments, PA14 WT and PA14 Transposon (Tn) mutants derived from a *P. aeruginosa* transposon-mutant library were also used [24,26,27].

*P. aeruginosa* strains were cultured as described previously [16]. In short, strains were grown in lysogene broth (LB) broth overnight at 37°C with shaking at 250 revolutions per minute (rpm). All overnight *P. aeruginosa* cultures (PA14 WT, PA14 Tn mutants, PAO1, AMT 0023–30, AA44, and the 7 clinical isolates from patient 6) were diluted in SCFM2 to an OD_590_ of 0.005, equivalent to 5 × 10^7^ CFU/mL, and then further diluted to reach a bacterial cell density of 5 × 10^5^ CFU/mL. SCFM2 was prepared as described previously [17], except that mucin was autoclaved instead of UV-sterilized to ensure reliable sterility. Bacterial suspensions were incubated for 48 h under microaerophilic conditions (5.5–12% O_2_) using Oxoid™ CampyGen™ Compact Sachet (CN0020C, Thermo Fisher Scientific) sealed with a plastic pouch to mimic oxygen-restricted conditions observed in pwCF with advanced lung disease [28]. The medium control (MC), i.e. SCFM2 without bacteria, was incubated in parallel. Afterward, the bacterial suspensions and the medium control were centrifuged (3500 rpm, 10 min) and the resulting supernatants were passed through a 0.22 µm filter to collect the cell-free supernatant. Supernatants were stored at −20°C without repeated freeze/thaw cycles.

Cell-free supernatants of PA14 Tn mutants were tested in the presence or absence of 0.5% (v/v) LasB inhibitor **4b**. This phosphonic acid inhibitor (C_13_H_16_F_3_NO_4_P, MW: 338.08 g/mol) was previously developed by Konstantinović et al. [29]. The compound was first dissolved in DMSO, and the final concentration was adjusted to 0.5% (v/v) to minimize potential solvent effects of DMSO on bacterial culture supernatants, as previously described [29].

### Quantification of *P. aeruginosa* virulence factors

#### Pyoverdine measurement

Pyoverdine was quantified by transferring 200 μL of filtered, cell-free *P. aeruginosa* supernatant in duplicates into a flat-bottom 96-well plate. Absorbance was measured at 400 nm using the Victor® Nivo™ Multi-mode plate reader (Perkin Elmer, Shelton, CT, USA). PAO1 was used as a positive control.

#### Pyocyanin assay

Pyocyanin quantification was performed via the chloroform-HCl extraction method [30]. Briefly, 1.5 mL of filtered cell-free *P. aeruginosa* supernatant was mixed with 1.5 mL chloroform by vortexing, followed by centrifugation (5000 rpm, 10 min) to separate the aqueous and organic phases. The chloroform layer was then collected and mixed with 0.2 N HCl. After another round of vortexing and centrifugation, 300 μL of the resulting pink aqueous (HCl) phase was transferred to a flat-bottom 96 well-plate, and absorbance was measured at 520 nm with the EnVision Multilabel Plate Reader (Perkin Elmer, Shelton, CT, USA). In parallel, the chloroform-HCl extraction method was applied to the supernatant of the medium control (i.e. SCFM2 alone) and AMT 0023–30, which served as the negative and positive controls, respectively.

#### Rhamnolipid quantification

Semi-quantitative detection of rhamnolipid production was performed using agar plates containing cetyltrimethylammonium bromide (CTAB) and methylene blue (MB) [31–33]. Minimal medium agar plates were supplemented with 0.2 g/L CTAB and 5 mg/L MB. Holes were created into the plates and inoculated with 100 μL of a 5 × 10^7^ CFU/mL bacterial suspension in SCFM2. Plates were incubated at 37°C under microaerophilic conditions (3% O_2_, 5% CO_2_, and 92% N_2_) using a hypoxia chamber (Bactrox, Sheldon manufacturing Inc., Cornelius, OR, US) for 48 h. Halo diameters surrounding the holes were measured to quantify rhamnolipid production. The medium control (100 μL) and 100 mM Tween-80 (100 μL) were used as the negative and positive controls, respectively.

#### Endotoxin quantification

Endotoxin levels in the filtered supernatants were measured using the Pierce Chromogenic Endotoxin Quant Kit (Thermo Fisher Scientific) following manufacturer’s instructions. Lipopolysaccharide (LPS) from *P. aeruginosa* serotype 10 (1 mg/mL; Merck, Darmstadt, Germany) was used as a positive control. Samples were diluted with Endotoxin-Free Water to achieve reading within the linear area of the standard curve (0.1–1.0 EU/mL), specifically a dilution factor of 10^5^ was used for bacterial cell-free supernatants and 5 × 10^6^ for the positive control. Each biological replicate was analyzed in two technical replicates.

#### Proteolytic and elastolytic activity assays

The proteolytic activity was assessed using the azocasein assay, while elastolytic activity was measured with the elastin-Congo red assay. Both assays were performed as described previously [16], with minor modifications. For each assay, 250 μL azocasein solution or elastin-Congo red suspension was combined with 250 μL of GTSF-2 medium containing 40 % (v/v) filtered cell-free bacterial supernatant, with and without the addition of 0.5% (v/v) component **4b** (final concentration 50 μM).

For the azocasein assay, samples were incubated at 37°C for 1 h under shaking conditions (250 rpm). The reaction was stopped by adding 62.5 μL of 10% (w/v) trichloroacetic acid, followed by centrifugation at 13,000 rpm for 15 min. From the resulting supernatant, 100 μL was transferred to a flat-bottom 96-well plate and mixed with 100 μL 625 nM NaOH.

For the elastin-Congo red assay, samples were incubated at 37°C for 24 h under shaking conditions (250 rpm), followed by centrifugation at 13,000 rpm for 15 min. From the resulting supernatant, 200 μL was transferred to a flat-bottom 96-well plate. To avoid saturated absorbance reading, the supernatant was diluted in Milli-Q (MQ) water when necessary.

Finally, the OD was measured at 420 nm and 492 nm for the azocasein and elastin-Congo red assays, respectively, using the Victor® Nivo™ Multi-mode plate reader (Perkin Elmer). The same procedure was applied to the medium control mixed with either azocasein solution or elastin-Congo red suspension, which served as the negative control. Two technical replicates were performed for each biological replicate.

### Arbitrary PCR and sequencing to confirm the identity of transposon mutant

Arbitrary polymerase chain reaction (PCR) and sequencing were performed as described previously [24,26,27]. A culture derived from a purified *P. aeruginosa* PA14 Tn mutant colony was grown statically in 96-well plates containing 280 µL LB supplemented with 15 µg/mL gentamicin per well at 37°C for approximately 40 h. Following incubation, 70 µL of culture was transferred to PCR tubes and stored at −20°C. For DNA extraction, samples were thawed, lysed at 99°C for 10 min, and centrifuged to pellet cell debris (3,500 rpm, 5 min). For the first round of arbitrary PCR (ARB1), 3 µL of lysate was used as template, and amplification was performed with the transposon-specific primer PMFLGM.GB-3a and arbitrary primer ARB1D (Table 1), and the Q5® Hot Start High-Fidelity 2X Master Mix (New England Biolabs, Ipswich, MA USA) according to manufacturer’s instructions. Thermocycling conditions were as follows: 95°C for 5 min; 30 cycles of 95°C for 30 s, 47°C for 45 s, and 72°C for 1 min; followed by a final extension at 72°C for 5 min. For the second round of arbitrary PCR (ARB2), 5 µL of ARB1 reaction was used as template. Reactions were carried out with the transposon-specific primer PMFLGM.GB-2a and arbitrary primer ARB2A (Table 1), and the Q5® Hot Start High-Fidelity 2X Master Mix under the following conditions: 40 cycles of 95°C for 30 s, 45°C for 30 s and 72°C for 1 min; followed by a final extension at 72°C for 5 min. PCR products were purified by mixing 5 μL ARB2 reaction with 2 μL ExoSAP-IT reagent (Thermo Fisher Scientific) and processed according to the manufacturer’s protocol. Sanger sequencing was performed after mixing the purified products (10 ng/μL) with the sequencing primer (5 μM) (Table 1; LightRun Tube Service; Eurofins Genomics). The resulting sequences were then queried against the *P. aeruginosa* UCBPP-PA14 genome (taxid:208963) using the BLASTx algorithm implemented through the NCBI Basic Local Alignment Search Tool [34–36].

**Table 1.** Sequences of used primer pairs.

	Primer	Sequence
ARB1	PMFLGM.GB-3a	5”-TACAGTTTACGAACCGAACAGGC-3”
	ARB1D	5”-GGCCAGGCCTGCAGATGATGNNNNNNNNNNGTAT-3”
ARB2	PMFLGM.GB-2a	5”-TGTCAACTGGGTTCGTGCCTTCATCCG-3”
	ARB2A	5”-GGCCAGGCCTGCAGATGATG-3”
Sequencing	PMFLGM.GB-4a	5”-GACCGAGATAGGGTTGAGTG-3”

### Three-dimensional lung epithelial cell culture model

The human adenocarcinomic alveolar epithelial cell line A549 (ATCC, CCL185) was cultured as an organotypic 3D cell culture model using a rotating wall vessel (RWV) bioreactor, as described previously [20,21]. Briefly, A549 cells were maintained as monolayers in T75 flasks using GTSF-2 medium (Hyclone™, Logan, UT, USA) supplemented with 1.5 g/L sodium bicarbonate (Sigma-Aldrich), 10% heat-inactivated fetal bovine serum (FBS) (Thermo Fisher Scientific), 2.5 mg/L insulin-transferrin-sodium selenite (Sigma-Aldrich) and 1% penicillin-streptomycin (with a stock concentration of 10,000 units/mL penicillin and 10 mg/mL streptomycin; Sigma-Aldrich). Upon reaching confluence, cells were washed with Hank’s Balanced Salt Solution (HBSS) (Life Technologies, Carlsbad, CA, USA) and dissociated using 0.25% trypsin-EDTA (Thermo Fisher Scientific). Cell counts and viability were assessed via trypan blue (0.4%, Sigma Aldrich) using a hemocytometer. A suspension containing 2 × 10^6^ viable cells in supplemented GTSF-2 medium was combined with 0.25 g porcine-skin collagen-coated dextran beads (Cytodex®-3 microcarrier beads, Cytiva, Marlborough, MA, USA), transferred to RWV bioreactors – first in a slow turning lateral vessel (STLV) and after 1 week in a high-aspect rotating vessel (HARV) (Synthecon, Houston, TX, USA) – and maintained for 11–14 days to allow the formation of differentiated 3D lung epithelial aggregates. All cultures were incubated at 37°C with 5% CO_2_.

### *In vitro* cell-exposure assay

3D A549 aggregates were transferred to a flat-bottom 96-well plate at a density of 2.5 × 10^5^ cells per well in 150 µL GTSF-2 medium without FBS and antibiotics. Host cells were exposed to 100 µL of either cell-free supernatant from *P. aeruginosa* cultures or filtered medium control (SCFM2) for 4 h at 37°C under microaerophilic conditions (3% O_2_, 5% CO_2_ and 92% N_2_) using a hypoxia chamber (Bactrox, Sheldon manufacturing Inc.). The exposure experiment using cell-free supernatant of PA14 Tn mutant was performed in the absence or presence of 0.5% (v/v) component **4b** (final concentration 50 μM). As a positive pro-inflammatory control, 25 μL of supernatant of *P. aeruginosa* strain AA44 was used. Following incubation, the conditioned medium (i.e. the supernatant fraction from the 3D lung cells after exposure to bacterial cell-free supernatant) was collected and stored at −20°C until further analyses, avoiding repeated freeze/thaw cycles.

### Cytotoxicity assay

Previous work demonstrated that *P. aeruginosa* protease production interferes with conventional lactate dehydrogenase (LDH) quantification [37]. To overcome this, a modified protocol was applied [37]. In this approach, LDH release from the viable cell fraction remaining adherent to the microcarrier beads at the end of the exposure experiment is measured as an indicator of cell viability. Briefly, cultures were rinsed twice with HBSS, and adherent cells were lysed with 1% Triton X-100 (Sigma-Aldrich). LDH activity was subsequently quantified using the Lactate Dehydrogenase Activity Assay Kit (Sigma-Aldrich) according to the manufacturer’s instructions.

### Inflammatory marker quantification

For *in vitro* cell exposure assays, Interleukin (IL)-8 release was measured in the conditioned medium using the Human ELISA MAX^TM^ Standard Set (Biolegend) according to the manufacturer’s instructions. Samples were diluted in GTSF-2 medium without FBS and antibiotics.

### Proteomics analyses of the culture supernatants of clinical *P. aeruginosa* isolates grown in SCFM2

#### Comparative proteomics analysis & data visualization

**Figure 1.**
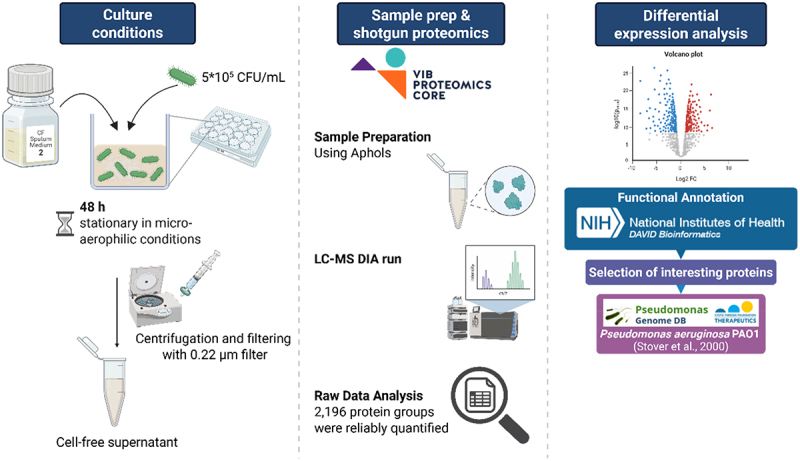
**Proteomics workflow:** Cell-free culture supernatants from clinical *P. aeruginosa* isolates grown in SCFM2 were collected through centrifugation and filtration. Proteomics sample preparation was performed using an amphipol-based protocol. Peptides were analyzed by LC-MS/MS, followed by raw spectral data analysis. Differential expression analysis was conducted by stratifying *P. aeruginosa* isolates into two groups, after which functional annotation was performed to identify proteins of interest. Created in BioRender. Wauters, M. (2026)*https://BioRender.com/7cm2xbx*.

Untargeted proteomics of cell-free supernatant from multiple *P. aeruginosa* CF isolates, including the isolates analyzed in this study, was previously performed in [16] and deposited in the ProteomeXchange Consortium via the PRIDE partner repository with the dataset identifier PXD065321. A new comparative proteomics data analysis was performed in the present study (comparing different isolate groups as previously), hereby comparing 5 pro- with 6 immunosuppressive *P. aeruginosa* CF isolates (listed in Table 2). Isolates were categorized into pro-inflammatory and immunosuppressive groups based on their statistically significant induction/reduction of IL-8 secretion by 3D lung epithelial cells which was investigated in [16] (Table 2 & Figure S1). Methodological details for sample preparation, liquid chromatography-tandem mass spectrometry (LC‑MS/MS) acquisition, and spectral data analysis are available in [16]. An overview of the workflow is presented in Figure 1.

**Table 2.** Sample groups according to isolate and inflammatory profile.

Sample name	Isolate	Inflammatory response	Number of samples
6/1 rep1-rep2-rep3-rep4	6/1	Immunosuppressive	4
12/1 rep1-rep2-rep3	12/1	Immunosuppressive	3
14/1 rep2-rep3-rep4	14/1	Immunosuppressive	3
15/1 rep2-rep3-rep4-rep1	15/1	Immunosuppressive	4
16/1 rep2-rep3-rep4-rep1	16/1	Immunosuppressive	4
19/1 rep2-rep3-rep4	19/1	Immunosuppressive	3
6/6 rep1-rep2-rep3-rep4	6/6	Pro-inflammatory	4
9/6 rep4-rep1-rep3	9/6	Pro-inflammatory	3
12/6 rep4-rep1-rep2	12/6	Pro-inflammatory	3
13/6 rep3-rep4-rep1	13/6	Pro-inflammatory	3
14/6 rep1-rep2-rep3	14/6	Pro-inflammatory	3

For differential expression analysis, missing values were imputed from a normal distribution using default parameters in Perseus [38]. Group comparisons were performed using a two-sample t-test with permutation-based false discovery rate (FDR) set at 5%.

#### Functional annotation

The gene names corresponding to the 605 significantly upregulated proteins (*p* < 0.05, fold change >2) in the pro-inflammatory isolate group were uploaded to the Database for Annotation, Visualization, and Integrated Discovery (DAVID) (search performed on 5 December 2024) [39,40]. Functional annotation was performed using *P. aeruginosa* PAO1 as the reference strain. Based on annotation terms linked to established virulence processes and/or PubMed text mining of the respective factors, 62 proteins were identified as being associated with virulence (Table S1).

#### Prediction of protein subcellular localization

The subcellular localization of mediators of interest was retrieved from the Pseudomonas Genome Database (version 22.1) with *P. aeruginosa* PAO1 (Stover *et al*.) as reference strain [41,42].

### Analysis of RNA-seq datasets of *P. aeruginosa* transcriptomes in CF sputum

Transcriptomic data for *P. aeruginosa*, including variance-stabilized (VST) normalized count data from sputum samples of pwCF, were obtained from the supplementary materials of Lewin *et al* [43]. To identify additional publicly available transcriptomic datasets, we accessed a comprehensive prerelease *P. aeruginosa* database (v0.1.0-beta) maintained by the Whiteley Lab at Georgia Institute for Technology (https://www.thewhiteleylab.com/database). This resource provides normalized gene expression data expressed as transcripts per million (TPM), along with the corresponding sample metadata. The downloaded input for the application included feature counts, data summaries, and metadata (dated: 18 July 2025). Filtered results of the transcriptomic dataset (from the original study Rossi *et al*. [44]; SRA: ERP106536) were saved as CSV files for subsequent analyses.

### Statistical analysis

ELISA data were processed using GainData® (Arigo Biolaboratories, available at https://www.arigobio.com/elisa-analysis). Standard curves were fitted using a four-parameter logistic regression model, and cytokine concentrations were calculated from absorbance values within the linear region of the curve.

Statistical analyses of virulence factor quantification, LDH cytotoxicity assay, cytokine concentrations of 3D aggregates exposed to PA14 WT and PA14 Tn mutants and correlation analysis were performed in GraphPad Prism (Version 10; https://www.graphpad.com). Data distribution was assessed using the Shapiro–Wilk test. Non-normally distributed data were analyzed using Mann–Whitney U or Kruskal–Wallis tests. To account for multiple comparisons, FDR correction was applied using the Benjamini–Hochberg procedure, with a significance set at 5% [45]. Paired data for proteolytic and elastolytic activities of the PA14 Tn mutants, measured in the absence or presence of compound **4b**, were analyzed using a mixed-effects model with restricted maximum likelihood (REML) estimation. Pearson correlation analysis was performed to assess the association between IL-8 release, endotoxin levels, and DksA abundance in bacterial cell-free supernatants. For each strain, at least three independent cell-free supernatants were collected for each assay. IL-8 release data were obtained from previously published experiments [16], in which 3D lung aggregates were exposed to bacterial supernatants. These data include the same bacterial isolates analyzed in the present study. DksA intensity was determined by LC-MS/MS, and endotoxin levels were measured using Pierce Chromogenic Endotoxin Quant Kit. For each strain, values were averaged across independent experiments. Correlation analysis was performed using mean IL-8 release values and the corresponding mean DksA abundance or endotoxin levels per strain.

All experiments were performed in at least three biological replicates. Figure and data visualizations were generated using GraphPad Prism and R (Version 4.4.2), employing R packages such as ggplot2 [46,47].

## Results

### Identification of candidate pro-inflammatory mediators of *P. aeruginosa* using comparative proteomics analysis

In our previous study, we observed that the supernatant obtained from *P. aeruginosa* CF isolates cultured in SCFM2 induced a variable inflammatory response based on IL-8 secretion in an organotypic 3D lung cell culture model. In particular, some isolates showed a pro-inflammatory response, while others exhibited an immunosuppressive response (Figure S1). All isolates causing robust pro-inflammatory effects in the 3D lung model were obtained from a single sputum sample from an individual with CF with long-term (over 19 years) chronic *P. aeruginosa* infection, while isolates leading to an immunosuppressive response were derived from an individual with CF who had become chronically infected relatively recently (1 year). Our previous work showed that proteolytic and elastolytic activities (partially driven by the metalloprotease elastase B, LasB) mediate the immunosuppressive effect of CF isolates through cytokine degradation [16]. In the present study, we aimed to identify pro-inflammatory mediators produced by CF isolates with pro-inflammatory activity.

To this end, a dual strategy was employed: (1) investigating the potential involvement of well-known virulence factors – pyocyanin, pyoverdine, rhamnolipids, and endotoxins – using semi-quantitative assays; (2) performing a comparative proteomics analysis of a previously generated dataset to characterize differences between pro-inflammatory and immunosuppressive isolate groups [16].

First, for all pro-inflammatory CF isolates tested, no significant production of any of the four well-established virulence factors was detected (Figure S2A, S2B, S2C, and S2D). Some CF isolates showed endotoxin levels of up to approximately 20,000 EU/mL, which, nevertheless, was not statistically significantly different from the medium control (SCFM2 alone) (Figure S2D). Furthermore, no significant correlation was observed between the endotoxin concentration in the cell-free supernatants and IL-8 release (Spearman’s correlation coefficient *r* = 0.0958; *p* = 0.8247; Figure S2E). Hence, well-known virulence factors of *P. aeruginosa* are likely not responsible for the observed pro-inflammatory effect.

**Figure 2.**
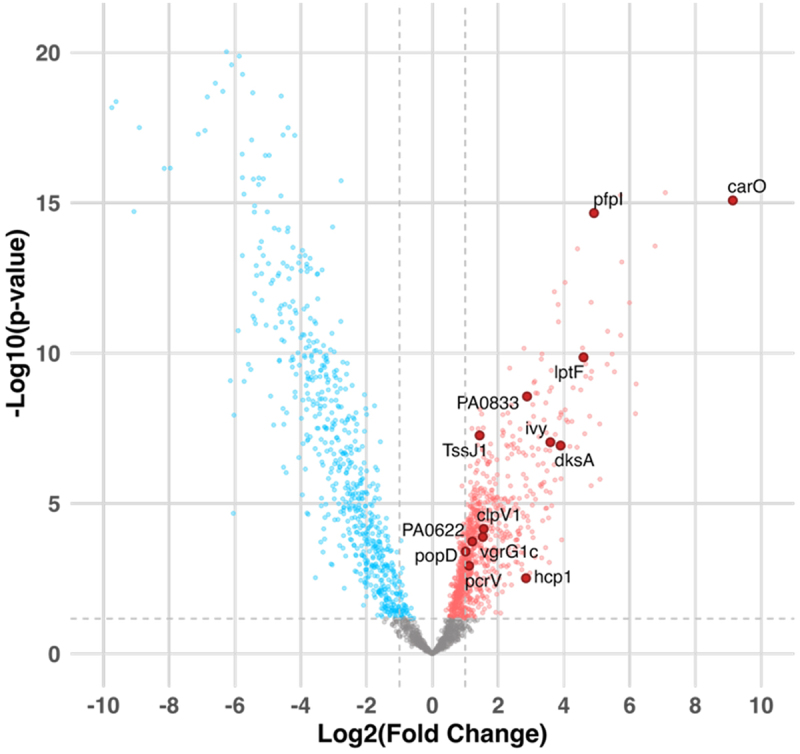
**Volcano plot with differentially expressed proteins of interest in the pro-inflammatory isolate group:** A volcano plot was generated to display the differences in protein abundance between the pro- and immunosuppressive isolate group. The difference is represented as log_2_(Fold change), plotted against -log_10_(*p*-values) derived from a two-sample t-test. Symbols corresponding to proteins of interest are enlarged and labeled. The horizontal dashed line indicates the significance threshold, determined using a permutation-based FDR correction of 5%.

Secondly, to identify previously unrecognized pro-inflammatory mediators of *P. aeruginosa*, we performed a comparative analysis of shotgun proteomics data from supernatants of CF isolates belonging to each of the inflammatory groups, after culturing in SCFM2 [16]. Data analysis was performed on 11 clinical isolates, classified into 6 immunosuppressive isolates and 5 pro-inflammatory isolates (Table 2). In total, 2,196 protein groups were quantified. A two-sample t-test between the pro-inflammatory and immunosuppressive isolate group was performed using a permutation-based FDR of 5% to correct for multiple testing. The results are presented in the volcano plot shown in Figure 2. Of the 1,236 significantly differentially expressed proteins (*p* < 0.05, fold change > |2|), a total of 605 were upregulated in the pro-inflammatory isolates group, while 631 proteins were downregulated (Table S2 and S3, respectively). The gene names corresponding to the 605 upregulated proteins in the pro-inflammatory isolate group were uploaded to DAVID to perform functional annotation. Based on annotation terms linked to established virulence processes, 62 proteins were identified as being associated with virulence (Table S1). Additionally, we observed a downregulation of multiple extracellular proteases – including LasB, alkaline protease A (AprA), and protease IV (PrpL) – alongside several proteins associated with Type IV pilus (PilV, PilW, PilY1, PilY2, and PilE) and flagellum-dependent motility (FliC, FlgK, EstA, FlgE, and CheZ) (Figure S3).

As a next step, we selected candidate pro-inflammatory mediators from the proteins upregulated in the pro-inflammatory isolate group for downstream *in vitro* validation of inflammatory activity. Candidates were selected based on functional annotation or PubMed text-mining evidence linking them to virulence, and/or strong upregulation (fold change > 5), as well as the availability of corresponding *P. aeruginosa* PA14 mutants in a Tn mutant library. Notably, the genes corresponding to some of the candidate pro-inflammatory mediators were not inactivated in the Tn mutant library or did not pass the arbitrary PCR quality control. In total, 13 mediators meeting these criteria were selected for further analysis (Figure 2; Table 3).

**Table 3.** **Selection of candidate pro-inflammatory proteins:** Subcellular localization is available in the Pseudomonas Genome database (https://www.pseudomonas.com/). Differential expression analysis between the pro-inflammatory and immunosuppressive isolate group was assessed using a two-sample t-test with multiple testing correction applied via a permutation-based FDR of 5%. Log_2_(Fold change) values are reported.

Gene	PA Locus	Log_2_(Fold Change)	Gene Name	Function and their association with virulence and/or inflammation (if available)	Localization	Ref.
carO	PA0320	9.137	Calcium-regulated OB-fold protein CarO	Maintaining intracellular Ca^2 +^ homeostasis	Unknown	[48]
dksA	PA4723	3.899	Suppressor protein DksA	Transcriptional regulator, stringent response modulator	Cytoplasmic	[49–53]
ivy	PA3902	3.588	Hypothetical protein	Inhibitor of lytic transglycosylase activity and vertebrate lysozyme	Periplasmic	[54,55]
vgrG1c	PA2685	1.535	Type VI secretion system spike protein VgrG1c	Secretion machinery protein of the contractile tail tube complex from the T6SS	Cytoplasmic	[56]
pcrV	PA1706	1.117	Type III secretion protein PcrV	Needle tip protein of the syringe-like injectisome T3SS	Extracellular	[57,58]
TssJ1	PA0080	1.438	Hypothetical protein	The T6SS consists of a membrane-anchored apparatus containing the outer membrane lipoprotein TssJ	Cytoplasmic MembraneOMV	[59]
clpV1	PA0090	1.560	Secretion protein ClpV1	an AAA + ATPase, which supplies energy to the T6SS. Its loss completely abolishes secretion activity	Cytoplasmic	[60]
hcp1	PA0085	2.847	Protein secretion apparatus assembly protein	Secretion machinery protein of the contractile tail tube complex from the T6SS	Extracellular	[56,57]
popD	PA1709	1.012	Translocator outer membrane protein PopD	PopD contributes to the formation of a translocation pore in the host cell membrane by the T3SS	Extracellular	[61]
lptF	PA3692	4.596	Lipotoxin F	Outer membrane protein that is highly expressed in mucoid *P. aeruginosa* isolates from chronic CF infections, survival factor, stimulates inflammatory responses, promising vaccine candidate	Outer Membrane OMV	[62–64]
PA0833	PA0833	2.879	OmpA-like domain-containing protein	Outer membrane protein, promising vaccine candidate	Outer Membrane OMV	[65,66]
pfpI	PA0355	4.915	Protease PfpI	Intracellular protease associated with antibiotic susceptibility, swarming motility, and biofilm formation	Cytoplasmic	[67]
PA0622	PA0622	1.215	Probable bacteriophage protein	A protein of unknown function that is highly abundant in OMVs from *P. aeruginosa* biofilms	Unknown OMV	[68–70]

Abbreviations: Outer Membrane Vesicle (OMV), Type III secretion system (T3SS), Type IV secretion system (T6SS).

### *In vitro* validation of candidate pro-inflammatory mediators

To evaluate the role of the selected mediators in modulating host inflammatory responses *in vitro*, IL-8 release was measured in an organotypic 3D lung cell culture model following exposure to cell-free supernatants from PA14 Tn mutant strains deficient in each mediator.

*P. aeruginosa* PA14 WT is known for high LasB production [29], and both the WT and Tn mutants exhibit high proteolytic and elastolytic activity (Figure S4A & S4B). A strong downregulation of LasB was observed in the pro-inflammatory isolates group, consistent with previous observations (Figure S3) [16]. Therefore, we inhibited LasB activity in the cell-free supernatants of the PA14 Tn mutant strains by addition of the LasB-specific phosphonic acid derivative **4b** [29] and subsequently quantified IL-8 release by the 3D lung aggregates.

**Figure 3.**
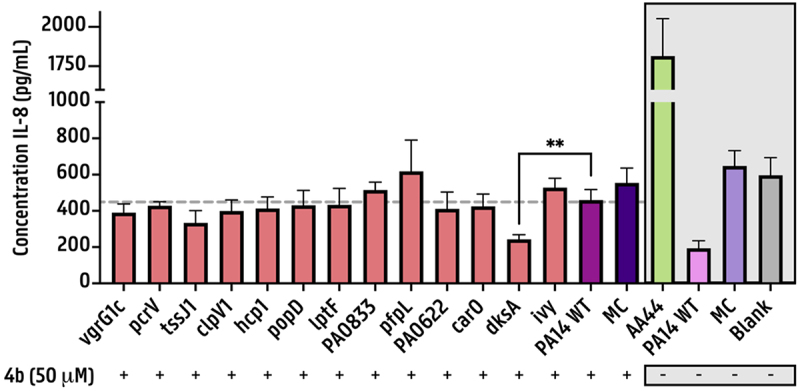
**Inflammatory response triggered by cell-free supernatants of PA14 Tn mutants, evaluated in the presence of LasB inhibition:** IL-8 release from an organotypic 3D lung cell culture model following 4h exposure to 40% (v/v) *P. aeruginosa* cell-free supernatants, in the presence of 50 μM LasB inhibitor.

Initially, the activity of compound **4b** was validated in our experimental setup using a dose–response study. Mixed-effects modeling with REML estimation revealed a significant inhibitory effect of compound **4b** (50 μM) on both proteolytic and elastolytic activities of the selected PA14 transposon mutants (*p* < 0.001 for each activity; Figure S4A & S4B). Treatment with the inhibitor resulted in a significant increase in IL-8 release by 3D lung aggregates exposed to PA14 Tn mutant cell-free supernatants, indicating restored cytokine activity due to reduced LasB-mediated degradation (Figure S5). Comparative analysis of IL-8 release induced by each Tn mutant strain and the PA14 WT demonstrated a significant anti-inflammatory effect only for PA14 dksA:Tn but not for other mutants (Figure 3).

Cell-viability assays were conducted to determine whether potential cytotoxic effects contributed to the observed results. Neither the bacterial supernatants nor the compound alone or in combination caused significant cytotoxicity in any experimental condition (Figure S6A & S6B).

### DksA-dependent regulation of virulence factors and the proteome

A significant correlation was observed between DksA abundance in the cell-free supernatants (Figure S7A) and IL-8 release (Spearman’s correlation coefficient *r* = 0.83; *p* = 0.0047; Figure S7B), indicating that higher extracellular levels of DksA were associated with stronger pro-inflammatory responses. To further assess the reduced inflammatory activity of PA14 dksA:Tn, we examined whether a similar effect could be detected in IL-6 release (Figure S8). Overall, IL-6 production by the 3D lung aggregates in response to the WT strain remained low, and no decrease in IL-6 levels was observed for PA14 dksA:Tn compared to the WT under **4b**-treated conditions. Furthermore, we assessed key pro-inflammatory virulence factors, specifically rhamnolipid production and proteolytic activity, as decreases in both have been linked to dksA mutations [49]. While no statistically significant differences were observed for the tested virulence factors, a clear reduction in rhamnolipid production was observed in PA14 dksA:Tn as compared to the wild-type strain (Figure S9A, S9B & S9C).

Given that DksA is a global transcriptional regulator influencing over 1,500 genes [52], we next examined the subset of DksA-regulated proteins that were differentially expressed exhibiting a fold change greater than |2| (as identified by our comparative proteomics analysis). This approach revealed 384 DksA-regulated proteins in our dataset (Figure S10 and Table S4), including several of the candidate pro-inflammatory proteins: CarO, PcrV, PopD, LptF, and PfpI (Table 3). Additionally, we identified multiple virulence-associated proteins, such as components of flagellar assembly (FlgE, FlgK, FliC), and type IV pilus biogenesis (pilV, pilW, pilY1, pilY2, and pilE), highlighting the wide impact of DksA on pathogenicity-related processes in *P. aeruginosa*.

### Validating the expression of *dksA* and other selected mediators in human CF sputum

**Figure 4.**
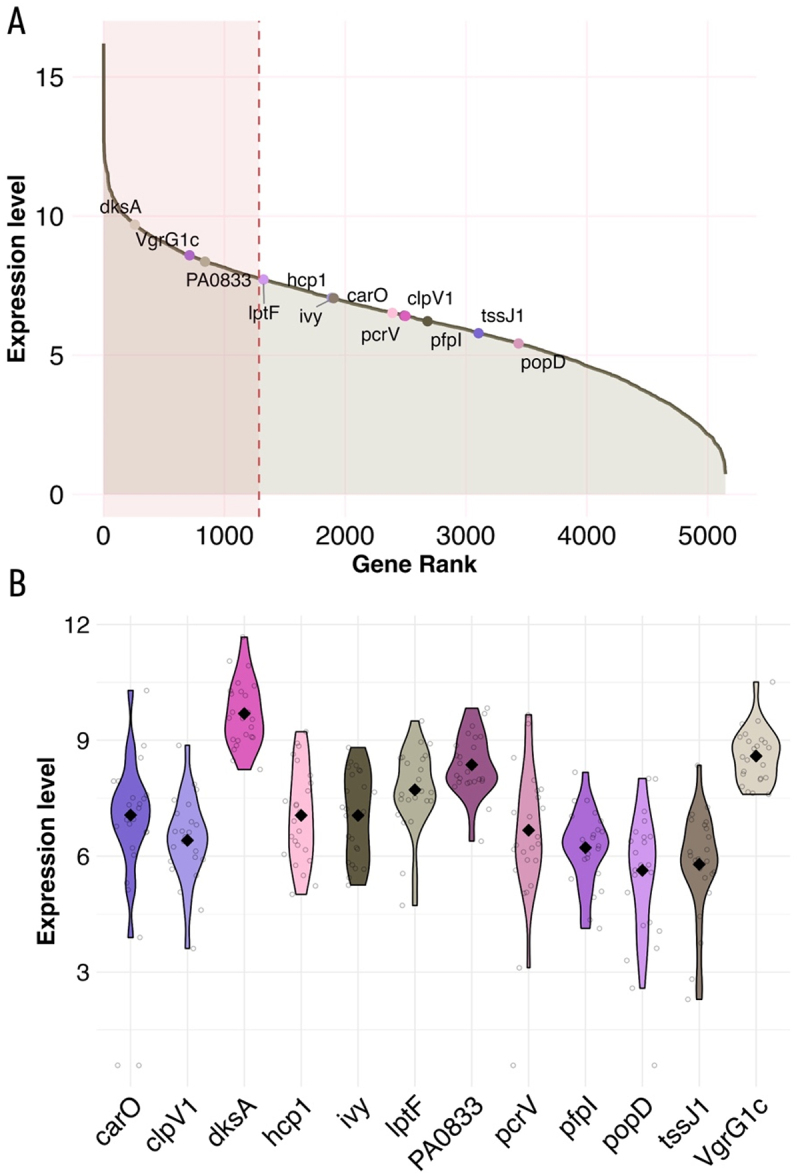
**Expression profile of mediators of interest across 24** ***P.** **aeruginosa*** **transcriptomes derived from CF sputum samples (data from Lewin *et*** ***al***. [43]): (A) Cumulative gene expression plot displaying genes ranked by their average expression (normalized count data) across the 24 CF sputum-derived *P. aeruginosa* transcriptomes. Proteins of interest are labeled, and the top quartile (25%) of most highly expressed genes is highlighted with a transparent red box; (B) Violin plots depicting the distribution and variability of expression levels for selected mediators across all 24 sam*ples with each violin representing one gene. Samples with absent expression (zero counts) were represented as dots at baseline and excluded from the violin plot visualization.*

Next, we evaluated the relevance of our findings for the CF population, by assessing the expression of *P. aeruginosa* pro-inflammatory genes in sputum of pwCF. Initially, we leveraged a transcriptomic dataset consisting of 24 *P. aeruginosa* sputum transcriptomes collected from 21 pwCF at two CF clinics, one in Copenhagen, Denmark, and the other in Atlanta, Georgia, USA, previously reported by Lewin *et* *al.* [43]. Genes were ranked by expression across CF sputum samples to identify those most highly expressed (Figure 4(A,B)). Notably, from our 13 selected mediators, *dksA, vgrG1c,* and *PA0833* ranked among the top 25% expressed genes. Normalized count data for *PA0622* was absent from the dataset as Lewin *et* *al.* excluded this gene due to its expression in only 14 of the 24 sputum samples. With the exception of *carO* (detected in 90% of samples), *pcrV,* and *popD* (each detected in 95%), all other mediators were expressed in 100% of the sputum transcriptomes [43].

In order to further investigate *P. aeruginosa* gene expression of the mediators in pwCF, we leveraged additional transcriptomic data using the *P. aeruginosa* database (https://www.thewhiteleylab.com/database). Data were leveraged from nine *P. aeruginosa* transcriptomes derived from CF sputum samples belonging to four different pwCF at different timepoints (originally derived from Rossi *et al* [44]). These samples were collected from Copenhagen, Denmark, and all four pwCF were chronically infected with *P.* *aeruginosa* DK01 and/or DK02 lineage for more than 30 years. The expression profiles of mediators of interest across all samples were visualized (Figure S12A and S12B), and *dksA, VgrG1c, PA0833, carO,* and *lptF* ranked among the top 25% expressed genes.

Given the confirmed role of DksA in the pro-inflammatory activity of *P. aeruginosa* (Figure 3), we further explored *dksA* expression in both transcriptomic datasets derived from CF sputum samples. Since *P. aeruginosa* encodes two functional *dksA* paralogs, we extended our analysis by including *dksA2*. Both *dksA1* (synonym: *dksA*) and *dksA2* regulators were among the top 25% of expressed genes in all sputum samples (Figure S11A, S11B, S12C, and S12D; data from Lewin *et* *al.* [43] and Rossi *et* *al.* [44], respectively).

## Discussion

### The role of DksA in *P. aeruginosa*-driven inflammation

Despite downregulation of well-known virulence factors, late-stage CF isolates of *P. aeruginosa* have been shown to elicit strong pro-inflammatory cytokine responses *in vitro*, *in vivo* using animal models and in the lungs of pwCF [11–13]. In this study, we aimed to identify previously unrecognized bacterial mediators sustaining this persistent inflammation by analyzing multiple pro-inflammatory isolates from an individual with CF with over 19 years of chronic *P. aeruginosa* infection.

The presence of major virulence factors, including pyocyanin, pyoverdine, rhamnolipids, and LPS, was minimal in the secretome of pro-inflammatory isolates, suggesting they do not contribute to the observed phenotype. Although other virulence factors were not directly evaluated, comparative proteomics analysis revealed a downregulation of multiple extracellular proteases, in line with reduced proteolytic activity measured in our previous study [16], along with proteins associated with type IV pilus and flagellum-dependent motility. This analysis guided the selection of 13 upregulated mediators from the pro-inflammatory isolates for further investigation. Using *P. aeruginosa* PA14 Tn mutants for validation, we found that the transcriptional regulator *dksA* was involved in the IL-8 pro-inflammatory response of this pathogen.

Indeed, DksA was a notable finding in the proteome of the cell-free supernatants, given its known intracellular localization. Its extracellular presence is most likely due to cell lysis, although incorporation into outer membrane vesicles (OMVs) or release during cytoplasmic leakage associated with OMV formation is also possible. Additionally, several cytosolic proteins are known to perform additional extracellular functions, such as promoting biofilm formation or enhancing virulence [68,71]. We were unable to directly assess the activity of extracellular DksA, and it thus remains unclear whether DksA has a direct pro-inflammatory effect on epithelial cells, which requires further investigation. We hypothesize that DksA indirectly contributes to IL-8 induction by regulating the production of specific secreted factor(s). Indeed, DksA is known to transcriptionally regulate hundreds of genes, including quorum-sensing-dependent virulence genes (e.g. encoding rhamnolipids and elastase), genes involved in tolerance to H_2_O_2_-induced oxidative stress, and protection against macrophage-mediated killing [52,53]. These diverse regulatory roles suggest that DksA may influence host inflammatory responses through modulation of the bacterial secretome rather than through direct extracellular activity. A recent study by Weimann *et al.* [72] demonstrated that intracellular survival within CF macrophages is facilitated by DksA1. Moreover, the authors reported that both the expression of the stringent response modulator *dksA1* and the activation of its associated regulon were linked to CF-specific adaptation of *P. aeruginosa* [72].

We confirmed in the PA14 dksA:Tn mutant that rhamnolipid production was controlled by DksA. Since the pro-inflammatory CF isolates tested in the present study showed no detectable rhamnolipid production (which is often observed for chronic isolates [73]), the observed immunomodulation by DksA is likely not mediated by these molecules. Furthermore, in this study, we focused on the pro-inflammatory cytokine IL-8, a key neutrophil chemoattractant that drives the pronounced infiltration of neutrophils characteristic of chronic *P. aeruginosa* infection in pwCF [4]. Indeed, other cytokines, including IL-6, are poorly expressed under the experimental conditions of the present study [16]. This could be explained by the presence of *P. aeruginosa* proteases, in addition to LasB, that degrade IL-6. In turn, cytokine degradation may result in the very low detectable levels observed following exposure to cell-free bacterial supernatants [16]. Further studies are needed to validate the role of DksA across a broader panel of cytokines relevant to the inflamed CF lung environment.

Interestingly, *P. aeruginosa* encodes two functional *dksA* paralogs, *dksA1* and *dksA2*, which are largely interchangeable but exhibit optimal activity under different environmental conditions [52]. The zinc-finger motif of zinc-dependent dksA1 becomes structurally unstable during zinc-depletion, whereas *dksA2* is exclusively expressed under zinc starvation [52,74,75]. Transcriptomic analyses of *P. aeruginosa* in CF sputum revealed high expression of both paralogs in our study. However, considering zinc starvation conditions in the CF lung environment, the biological relevance of increased *dksA1* expression under these conditions requires further clarification [43,44,76]. *Z*inc deprivation is a well-recognized host-defense mechanism, exacerbated in CF sputum by high levels of calprotectin, a neutrophil-derived zinc-chelating protein [76]. When comparing *in vivo* transcriptomes of 12 CF sputum samples with *in vitro* stationary-phase LB cultures of *P. aeruginosa* PA14 and matched clinical isolates, Rossi *et al*. [44] observed strong induction of *dksA2* and repression of *dksA*1. These findings indicate that, during CF lung infection, zinc-independent *dksA2* predominates in mediating stress responses [44]. In the present study, proteomics analysis did not reveal DksA2 in the culture supernatant of the clinical CF isolates, likely because the isolates were cultured in SCFM2, which is not zinc-limited. Indeed, when zinc limitation is established in SCFM2 by chelation with calprotectin, *dksA2* becomes expressed [43]. Hence, while DksA1 was likely involved in the pro-inflammatory response of *P. aeruginosa* in the model systems used in our study, further investigation is needed to define the specific roles of DksA1 and DksA2 and their downstream gene regulatory networks in the *P. aeruginosa*-induced inflammation in the CF lung environment.

### Insights into other candidate pro-inflammatory mediators

Furthermore, 12 other candidate mediators were explored for their role in the pro-inflammatory response, but the cell-free supernatant of each individual mediator PA14 Tn mutant did not significantly influence IL-8 release relative to the PA14 wild-type. Six mutants were associated with the T3SS or T6SS, which are virulence factors that operate via a one-step mechanism that injects toxic effector proteins into target cells [77,78]. Both secretion systems have been shown to trigger host inflammation by inducing cytokine production such as IL-6, IL-8, and IL-1β in A549 cells and macrophages [79–82]. Furthermore, immunization with a trivalent vaccine containing PcrV (T3SS), OprI, and Hcp1 (T6SS) proteins conferred protection in a murine *P. aeruginosa* pneumonia model [57], and a phase I/II trial in pwCF demonstrated that the anti-PcrV antibody KB001-A was effective in reducing sputum IL-8 levels [58]. However, in this study, none of the T3SS- or T6SS-related mutants induced altered inflammatory responses relative to the PA14 wild-type. A key difference between our study and previous work is that earlier studies infected cells with live bacteria, whereas we employed cell-free supernatants. Since the *P. aeruginosa* secretion systems are anchored to the outer membrane and require this association for activity, their activity is typically absent in cell-free supernatants. Nevertheless, *P. aeruginosa* secretes OMVs, which have been implicated in virulence-associated interactions with lung epithelial cells [83,84]. While the presence of T3SS or T6SS proteins in *P. aeruginosa* OMVs has not been definitively reported, T3SS effectors and translocon proteins have been identified in OMVs from other bacteria including *Salmonella enterica* and *Escherichia coli* O157:H7 [85–87].

Analyses of publicly accessible transcriptomic data of CF sputum revealed high expression of both *PA0833* and *lptF*. Notably, *lptF* (Lipotoxin F) is highly expressed in mucoid *P. aeruginosa* isolates from chronic CF infections [62] and, along with PA0833, is secreted via OMVs [41,42,70,88]. Both proteins have emerged as promising vaccine candidates. Immunization with PA0833 has been shown to protect against *P. aeruginosa* in a pneumonia mouse model [65,66], while LptF activated NF-κB in human respiratory epithelial cells via Toll-like receptor 2 [63]. Additionally, recent immunoinformatic approaches have designed an epitope-based peptide vaccine based on LptF that shows high stability and immunogenicity *in silico* [64]. Elucidating the specific contributions of both mediators to inflammation remains an interesting direction for future investigation.

## Conclusion

This study reveals considerable variability in the inflammatory profile of *P. aeruginosa* isolates from pwCF, with some retaining strong inflammatory activity despite the loss of well-known virulence traits. Culturing these isolates under physiologically relevant conditions uncovered mediators that could play a role in the persistent inflammation in the lungs of pwCF. Interestingly, these mediators were consistently and robustly expressed across all analyzed *P. aeruginosa* CF sputum transcriptomes. While DksA was the only mediator for which a pro-inflammatory role could be confirmed, it is likely that it may act in an additive or synergistic way with other identified mediators. Hence, the pro-inflammatory response is probably driven by multiple mediators in concert rather than a single factor. Future studies should elucidate potential interactions among mediators underlying the inflammatory response and identify those with the greatest promise as therapeutic targets to mitigate chronic inflammation in *P. aeruginosa* CF lung infections.

## Supplementary Material

Manuscript_DksA_Virulence_Resubmission_Supplem.docx

## Data Availability

Mass spectrometry-based proteomics from Wauters *et al.* [16] are openly available via the ProteomeXchange Consortium through the PRIDE partner repository under the dataset identifier PXD065321. This study used only fully anonymized, publicly available data released under Creative Commons licenses that permit reuse under specified conditions (e.g. non‑commercial use and/or no derivatives); therefore, no additional ethical approval was required. *P. aeruginosa* CF sputum transcriptome normalized count data from Lewin *et al.* [43] are available as Dataset S3 in the original publication and were released under the terms of the CC BY-NC-ND 4.0 license. *P. aeruginosa* CF sputum transcriptome normalized data from Rossi *et al.* [44] were obtained from the *P. aeruginosa* database (v0.1.0-beta; https://www.thewhiteleylab.com/database) and are released under the terms of the CC BY 4.0 license. The raw data that support the findings of this study are publicly available in Zenodo with DOI: 10.5281/zenodo.19369593 (https://doi.org/10.5281/zenodo.19369593), under the Ghent University Research Data Community [90].

## References

[1] Kerem E, Viviani L, Zolin A, et al. Factors associated with FEV1 decline in cystic fibrosis: analysis of the ECFS patient registry. Eur Respir J. 2013;43(1):125–16. 23598952 10.1183/09031936.00166412

[2] Robertson JK, Goldberg JB, Robertson JK, et al. The impact of cystic fibrosis transmembrane conductance regulator (CFTR) modulators on the pulmonary microbiota. Microbiology. 2025;171(4):001553. doi: 10.1099/mic.0.001553 40202901 PMC12282298

[3] Valladares KN, Jones LI, Barnes JW, et al. Highly effective modulator therapy: implications for the microbial landscape in cystic fibrosis. Int J Mol Sci. 2024;25(22):11865. doi: 10.3390/ijms252211865 39595943 PMC11594123

[4] Nickerson R, Thornton CS, Johnston B, et al. *Pseudomonas aeruginosa* in chronic lung disease: untangling the dysregulated host immune response. Front Immunol. 2024;15:1405376. doi: 10.3389/fimmu.2024.1405376 39015565 PMC11250099

[5] Cohen TS, Prince A. Cystic fibrosis: a mucosal immunodeficiency syndrome. Nat Med. 2012;18(4):509–519. doi: 10.1038/nm.2715 22481418 PMC3577071

[6] Letizia M, Diggle SP, Whiteley M. *Pseudomonas aeruginosa*: ecology, evolution, pathogenesis and antimicrobial susceptibility. Nat Rev Microbiol. 2025;23(11):701–717. doi: 10.1038/s41579-025-01193-8 40442328 PMC13064840

[7] Cystic Fibrosis Foundation. 2024 patient registry highlights. 2024. https://pr.ecfs.eu/annual-reports/.

[8] Zolin A, Adamoli A, Bakkeheim E, et al. European Cystic Fibrosis Society Patient Registry annual report 2023. European Cystic Fibrosis Society Patient; 2023.

[9] Faure E, Kwong K, Nguyen D. *Pseudomonas aeruginosa* in chronic lung infections: how to adapt within the host? Front Immunol. 2018;9:2416. doi: 10.3389/fimmu.2018.02416 30405616 PMC6204374

[10] Rossi E, La Rosa R, Ja B, et al. *Pseudomonas aeruginosa* adaptation and evolution in patients with cystic fibrosis. Nat Rev Microbiol. 2020;19(5):331–342. 33214718 10.1038/s41579-020-00477-5

[11] LaFayette SL, Houle D, Beaudoin T, et al. Cystic fibrosis-adapted *Pseudomonas aeruginosa* quorum sensing *lasR* mutants cause hyperinflammatory responses. Sci Adv. 2015;1(6):e1500199. 26457326 10.1126/sciadv.1500199 PMC4597794

[12] Bragonzi A, Paroni M, Nonis A, et al. *Pseudomonas aeruginosa* microevolution during cystic fibrosis lung infection establishes clones with adapted virulence. Am J Respir Crit Care Med. 2009;180(2):138–145. doi: 10.1164/rccm.200812-1943OC 19423715

[13] Phuong MS, Hernandez RE, Wolter DJ, et al. Impairment in inflammasome signaling by the chronic *Pseudomonas aeruginosa* isolates from cystic fibrosis patients results in an increase in inflammatory response. Cell Death Dis. 2021;12(241).doi:10.1038/s41419-021-03526-w PMC7933143 33664232

[14] Hennemann LC, LaFayette SL, Malet JK, et al. Lasr-deficient *Pseudomonas aeruginosa* variants increase airway epithelial MICAM-1 expression and enhance neutrophilic lung inflammation. PLOS Pathog. 2021;17(3):e1009375. doi: 10.1371/journal.ppat.1009375 33690714 PMC7984618

[15] Rossi E, Lausen M, Øbro NF, et al. Widespread alterations in systemic immune profile are linked to lung function heterogeneity and airway microbes in cystic fibrosis. J Cyst Fibros. 2024;23(5):885–895. doi: 10.1016/j.jcf.2024.04.015 38702223

[16] Wauters M, Van Den Bossche S, Grassi L, et al. Unraveling the immunosuppressive role of elastase B produced by cystic fibrosis isolates of *Pseudomonas aeruginosa* in an organotypic 3D lung epithelial cell model. Virulence. 2025;16(1). doi: 10.1080/21505594.2025.2581889 PMC12599356 41196696

[17] Turner KH, Wessel AK, Palmer GC, et al. Essential genome of *Pseudomonas aeruginosa* in cystic fibrosis sputum. Proc Natl Acad Sci USA. 2015;112(13):4110–4115. doi: 10.1073/pnas.1419677112 25775563 PMC4386324

[18] Cornforth DM, Diggle FL, Melvin JA, et al. Quantitative framework for model evaluation in microbiology research using *Pseudomonas aeruginosa* and cystic fibrosis infection as a test case. Mbio. 2020;11(1):e03042–19. doi: 10.1128/mBio.03042-19 31937646 PMC6960289

[19] Barrila J, Radtke AL, Crabbe A, et al. Organotypic 3D cell culture models: using the rotating wall vessel to study host-pathogen interactions. Nat Rev Microbiol. 2010;8(11):791–801. doi: 10.1038/nrmicro2423 20948552

[20] Crabbe A, Liu Y, Matthijs N, et al. Antimicrobial efficacy against *Pseudomonas aeruginosa* biofilm formation in a three-dimensional lung epithelial model and the influence of fetal bovine serum. Sci Rep. 2017;7:43321. doi: 10.1038/srep43321 28256611 PMC5335707

[21] Carterson AJ, Honer Zu Bentrup K, Ott CM, et al. A549 lung epithelial cells grown as three-dimensional aggregates: alternative tissue culture model for *Pseudomonas aeruginosa* pathogenesis. Infect Immun. 2005;73(2):1129–1140. doi: 10.1128/IAI.73.2.1129-1140.2005 15664956 PMC547019

[22] Van Den Bossche S, Abatih E, Grassi L, et al. Pooling isolates to address the diversity in antimicrobial susceptibility of *Pseudomonas aeruginosa* in cystic fibrosis. Microbiol Spectr. 2023;11(6):e0044923. doi: 10.1128/spectrum.00449-23 37982625 PMC10714813

[23] Cullen L, Weiser R, Olszak T, et al. Phenotypic characterization of an international *Pseudomonas aeruginosa* reference panel: strains of cystic fibrosis (CF) origin show less *in vivo* virulence than non-CF strains. Microbiology. 2015;161(10):1961–1977. doi: 10.1099/mic.0.000155 26253522

[24] Jacobs MA, Alwood A, Thaipisuttikul I, et al. Comprehensive transposon mutant library of *Pseudomonas aeruginosa*. Proc Natl Acad Sci USA. 2003;100(24):14339–14344. doi: 10.1073/pnas.2036282100 14617778 PMC283593

[25] Chandler CE, Horspool AM, Hill PJ, et al. Genomic and phenotypic diversity among ten laboratory isolates of *Pseudomonas aeruginosa* PAO1. J Bacteriol. 2018;201(5). doi: 10.1128/jb.00595-18 PMC6379574 30530517

[26] Liberati NT, Urbach JM, Miyata S, et al. An ordered, nonredundant library of *Pseudomonas aeruginosa* strain Pa14 transposon insertion mutants. Proc Natl Acad Sci USA. 2006;103(8):2833–2838. doi: 10.1073/pnas.0511100103 16477005 PMC1413827

[27] Liberati NT, Urbach JM, Miyata S, et al. Pa14 non-redundant transposon insertion mutant set (Pa14nr set) 2006 [Available from: https://pa14.mgh.harvard.edu/cgi-bin/pa14/home.cgi

[28] Worlitzsch D, Tarran R, Ulrich M, et al. Effects of reduced mucus oxygen concentration in airway *Pseudomonas* infections of cystic fibrosis patients. J Clin Invest. 2002;109(3):317–325. doi: 10.1172/JCI0213870 11827991 PMC150856

[29] Konstantinović J, Kany AM, Alhayek A, et al. Inhibitors of the elastase lasb for the treatment of *Pseudomonas aeruginosa* lung infections. ACS Cent Sci. 2023;9(12):2205–2215. doi: 10.1021/acscentsci.3c01102 38161367 PMC10755728

[30] Zhang Y, Sass A, Van Acker H, et al. Coumarin reduces virulence and biofilm formation in *Pseudomonas aeruginosa* by affecting quorum sensing, type III secretion and c-di-GMP levels. Front Microbiol. 2018;9:1952. doi: 10.3389/fmicb.2018.01952 30186266 PMC6110822

[31] Jiang J, Jin M, Li X, et al. Recent progress and trends in the analysis and identification of rhamnolipids. Appl Microbiol Biotechnol. 2020;104(19):8171–8186. doi: 10.1007/s00253-020-10841-3 32845366

[32] Pinzon NM, Ju LK. Improved detection of rhamnolipid production using agar plates containing methylene blue and cetyl trimethylammonium bromide. Biotechnol Lett. 2009;31(10):1583–1588. doi: 10.1007/s10529-009-0049-7 19547929

[33] Siegmund I, Wagner F. New method for detecting rhamnolipids excreted by *Pseudomonas* species during growth on mineral agar. Biotechnol Tech. 1991;5(4):265–268. doi: 10.1007/BF02438660

[34] Sayers, Eric J, Bolton. Database resources of the National Center for Biotechnology Information in 2025. Nucleic Acids Res. 2025;53(D1):D20–D29. doi: 10.1093/nar/gkae979 39526373 PMC11701734

[35] Altschul SF, Gish W, Miller W, et al. Basic local alignment search tool. J Mol Biol. 1990;215(3):403–410. doi: 10.1016/S0022-2836(05)80360-2 2231712

[36] Camacho C, Coulouris G, Avagyan V, et al. Blast+: architecture and applications. BMC Bioinf. 2009;10(1):421. doi: 10.1186/1471-2105-10-421 PMC2803857 20003500

[37] Van den Bossche S, Vandeplassche E, Ostyn L, et al. Bacterial interference with lactate dehydrogenase assay leads to an underestimation of cytotoxicity. Front Cell Infect Microbiol. 2020;10:494. doi: 10.3389/fcimb.2020.00494 33042868 PMC7523407

[38] Tyanova S, Cox J. Perseus: a bioinformatics platform for integrative analysis of proteomics. Methods Mol Biol. 2018;1711:133–148. 29344888 10.1007/978-1-4939-7493-1_7

[39] Huang DW, Sherman BT, Lempicki RA, et al. Systematic and integrative analysis of large gene lists using DAVID Bioinformatics Resources. Nat Protoc. 2008;4(1):44–57. 10.1038/nprot.2008.211 19131956

[40] Sherman BT, Hao M, Qiu J, et al. David: a web server for functional enrichment analysis and functional annotation of gene lists (2021 update). Nucleic Acids Res. 2022;50(W1):W216–W21. doi: 10.1093/nar/gkac194 35325185 PMC9252805

[41] Winsor G, Griffiths E, Lo R, et al. Enhanced annotations and features for comparing thousands of Pseudomonas genomes in the *Pseudomonas* genome database. Nucleic Acids Res. 2016;44(D1):D646–53. doi: 10.1093/nar/gkv1227 26578582 PMC4702867

[42] Stover C, Pham X, Erwin A, et al. Complete genome sequence of *Pseudomonas aeruginosa* PAO1, an opportunistic pathogen. Nature. 2000;406(6799):959–964. doi: 10.1038/35023079 10984043

[43] Lewin GR, Kapur A, Cornforth DM, et al. Application of a quantitative framework to improve the accuracy of a bacterial infection model. Proc Natl Acad Sci. 2023;120(19):e2221542120. doi: 10.1073/pnas.2221542120 37126703 PMC10175807

[44] Rossi E, Falcone M, Molin S, et al. High-resolution in situ transcriptomics of *Pseudomonas aeruginosa* unveils genotype independent patho-phenotypes in cystic fibrosis lungs. Nat Commun. 2018;9(1):3459. doi: 10.1038/s41467-018-05944-5 30150613 PMC6110831

[45] Benjamini Y, Hochberg Y. Controlling the false discovery rate: a practical and powerful approach to multiple testing. J R Stat Soc Ser B (Methodological). 1995;57(1):289–300. doi: 10.1111/j.2517-6161.1995.tb02031.x

[46] Team RC. R: a language and environment for statistical computing. Vienna, Austria: Foundation for Statistical Computing; 2024.

[47] Wickham H. Ggplot2: elegant graphics for data analysis. Springer-Verlag New York:USA. 2016.

[48] Guragain M, King MM, Williamson KS, et al. The *Pseudomonas aeruginosa* PAO1 two-component regulator CarSR regulates calcium homeostasis and calcium-induced virulence factor production through its regulatory targets CarO and CarP. J Bacteriol. 2016;198(6):951–963. doi: 10.1128/JB.00963-15 26755627 PMC4772601

[49] Jude F, Köhler T, Branny P, et al. Posttranscriptional control of quorum-sensing-dependent virulence genes by Dksa in *Pseudomonas aeruginosa*. J Bacteriol. 2003;185(12):3558–3566. 12775693 10.1128/JB.185.12.3558-3566.2003 PMC156223

[50] Min KB, Yoon SS. Transcriptome analysis reveals that the RNA polymerase–binding protein DksA1 has pleiotropic functions in *Pseudomonas aeruginosa*. J Biol Chem. 2020;295(12):3851–3864. doi: 10.1074/jbc.RA119.011692 32047111 PMC7086037

[51] Min KB, Hwang W, Lee K-M, et al. Chemical inhibitors of the conserved bacterial transcriptional regulator Dksa1 suppressed quorum sensing-mediated virulence of *Pseudomonas aeruginosa*. J Biol Chem. 2021;296:100576. doi: 10.1016/j.jbc.2021.100576 33757766 PMC8081920

[52] Fortuna A, Bähre H, Visca P, et al. The two *Pseudomonas aeruginosa* DksA stringent response proteins are largely interchangeable at the whole transcriptome level and in the control of virulence-related traits. Environ Microbiol. 2021;23(9):5487–5504. doi: 10.1111/1462-2920.15693 34327807

[53] Fortuna A, Collalto D, Schiaffi V, et al. The *Pseudomonas aeruginosa* Dksa1 protein is involved in H2O2 tolerance and within-macrophages survival and can be replaced by Dksa2. Sci Rep. 2022;12(1):10404. doi: 10.1038/s41598-022-14635-7 35729352 PMC9213440

[54] Abergel C, Monchois V, Byrne D, et al. Structure and evolution of the ivy protein family, unexpected lysozyme inhibitors in Gram-negative bacteria. Proc Natl Acad Sci. 2007;104(15):6394–6399. doi: 10.1073/pnas.0611019104 17405861 PMC1847508

[55] Clarke CA, Scheurwater EM, Clarke AJ. The vertebrate lysozyme inhibitor ivy functions to inhibit the activity of lytic transglycosylase. J Biol Chem. 2010;285(20):14843–14847. doi: 10.1074/jbc.C110.120931 20351104 PMC2865275

[56] Spínola-Amilibia M, Davó-Siguero I, Ruiz FM, et al. The structure of Vgrg1 from *Pseudomonas aeruginosa*, the needle tip of the bacterial type VI secretion system. Acta Crystallogr Sect D Struct Biol. 2016;72(1):22–33. doi: 10.1107/S2059798315021142 26894531

[57] Yang F, Gu J, Yang L, et al. Protective efficacy of the trivalent *Pseudomonas aeruginosa* vaccine candidate pcrV-OprI-Hcp1 in murine pneumonia and burn models. Sci Rep. 2017;7(1):3957. doi: 10.1038/s41598-017-04029-5 28638106 PMC5479855

[58] Milla CE, Chmiel JF, Accurso FJ, et al. Anti-pcrv antibody in cystic fibrosis: a novel approach targeting *Pseudomonas aeruginosa* airway infection. Pediatr Pulmonol. 2014;49(7):650–658. doi: 10.1002/ppul.22890 24019259 PMC4079258

[59] Robb CS, Assmus M, Nano FE, et al. Structure of the T6SS lipoprotein TssJ1 from *Pseudomonas aeruginosa*. Acta Crystallogr Sect F Struct Biol Crystallization Commun. 2013;69(6):607–610. doi: 10.1107/S1744309113012220 PMC3668576 23722835

[60] Chen L, Zou Y, Kronfl AA, et al. Type VI secretion system of *Pseudomonas aeruginosa* is associated with biofilm formation but not environmental adaptation. Microbiologyopen. 2020;9(3):e991. doi: 10.1002/mbo3.991 31961499 PMC7066461

[61] Tang Y, Romano FB, Breña M, et al. The *Pseudomonas aeruginosa* type III secretion translocator PopB assists the insertion of the PopD translocator into host cell membranes. J Biol Chem. 2018;293(23):8982–8993. doi: 10.1074/jbc.RA118.002766 29685888 PMC5995524

[62] Damron FH, Napper J, Teter MA, et al. Lipotoxin F of *Pseudomonas aeruginosa* is an algu-dependent and alginate-independent outer membrane protein involved in resistance to oxidative stress and adhesion to A549 human lung epithelia. Microbiology. 2009;155(4):1028–1038. doi: 10.1099/mic.0.025833-0 19332805 PMC2895231

[63] Firoved AM, Ornatowski W, Deretic V. Microarray analysis reveals induction of lipoprotein genes in mucoid *Pseudomonas aeruginosa*: implications for inflammation in cystic fibrosis. Infect Immun. 2004;72(9):5012–5018. doi: 10.1128/IAI.72.9.5012-5018.2004 15321993 PMC517412

[64] Mudipalli Elavarasu S, S K. Rational design of an epitope-centric vaccine against *Pseudomonas aeruginosa* using pangenomic insights and immunoinformatics approach. Front Immunol. 2025;16:1617251. 40959094 10.3389/fimmu.2025.1617251 PMC12434008

[65] Yang F, Gu J, Zou J, et al. Pa0833 is an ompa C-like protein that confers protection against *Pseudomonas aeruginosa* infection. Front Microbiol. 2018;9:1062. doi: 10.3389/fmicb.2018.01062 29875759 PMC5974059

[66] Zou J-T, Jing H-M, Yuan Y, et al. Pore-forming alpha-hemolysin efficiently improves the immunogenicity and protective efficacy of protein antigens. PLOS Pathog. 2021;17(7):e1009752. doi: 10.1371/journal.ppat.1009752 34288976 PMC8294524

[67] Fernández L, Breidenstein EBM, Song D, et al. Role of intracellular proteases in the antibiotic resistance, motility, and biofilm formation of *Pseudomonas aeruginosa*. Antimicrob Agents Chemother. 2012;56(2):1128–1132. doi: 10.1128/AAC.05336-11 22123702 PMC3264200

[68] Couto N, Schooling SR, Dutcher JR, et al. Proteome profiles of outer membrane vesicles and extracellular matrix of *Pseudomonas aeruginosa* biofilms. J Proteome Res. 2015;14(10):4207–4222. doi: 10.1021/acs.jproteome.5b00312 26303878

[69] Toyofuku M, Roschitzki B, Riedel K, et al. Identification of proteins associated with the *Pseudomonas aeruginosa* biofilm extracellular matrix. J Proteome Res. 2012;11(10):4906–4915. doi: 10.1021/pr300395j 22909304

[70] Choi DS, Kim DK, Choi SJ, et al. Proteomic analysis of outer membrane vesicles derived from *Pseudomonas aeruginosa*. Proteomics. 2011;11(16):3424–3429. doi: 10.1002/pmic.201000212 21751344

[71] Ebner P, Götz F. Bacterial excretion of cytoplasmic proteins (Ecp): occurrence, mechanism, and function. Trends Microbiol. 2019;27(2):176–187. doi: 10.1016/j.tim.2018.10.006 30442534

[72] Weimann A, Dinan AM, Ruis C, et al. Evolution and host-specific adaptation of *Pseudomonas aeruginosa*. Science. 2024;385(6704):eadi0908. doi: 10.1126/science.adi0908 38963857 PMC7618370

[73] Bjarnsholt T, Jensen PØ, Jakobsen TH, et al. Quorum sensing and virulence of *Pseudomonas aeruginosa* during lung infection of cystic fibrosis patients. PLOS ONE. 2010;5(4):e10115. doi: 10.1371/journal.pone.0010115 20404933 PMC2853559

[74] Blaby-Haas CE, Furman R, Rodionov DA, et al. Role of a Zn-independent DksA in Zn homeostasis and stringent response. Mol Microbiol. 2011;79(3):700–715. doi: 10.1111/j.1365-2958.2010.07475.x 21255113 PMC3076637

[75] Furman R, Biswas T, Danhart EM, et al. Dksa2, a zinc-independent structural analog of the transcription factor Dksa. FEBS Lett. 2013;587(6):614–619. doi: 10.1016/j.febslet.2013.01.073 23416301 PMC5525025

[76] Mastropasqua MC, Lamont I, Martin LW, et al. Efficient zinc uptake is critical for the ability of *Pseudomonas aeruginosa* to express virulence traits and colonize the human lung. J Trace Elem Med Biol. 2018;48:74–80. doi: 10.1016/j.jtemb.2018.03.009 29773197

[77] Colautti J, Kelly SD, Whitney JC. Specialized killing across the domains of life by the type VI secretion systems of *Pseudomonas aeruginosa*. Biochem J. 2025;482(1):1–15. doi: 10.1042/BCJ20230240 PMC12133291 39774785

[78] Hauser AR. The type III secretion system of *Pseudomonas aeruginosa*: infection by injection. Nat Rev Microbiol. 2009;7(9):654–665. doi: 10.1038/nrmicro2199 19680249 PMC2766515

[79] Galle M, Schotte P, Haegman M, et al. The *Pseudomonas aeruginosa* type III secretion system plays a dual role in the regulation of caspase-1 mediated IL-1beta maturation. J Cell Mol Med. 2008;12(5A):1767–1776. 18081695 10.1111/j.1582-4934.2007.00190.x PMC3918092

[80] Miao EA, Ernst RK, Dors M, et al. *Pseudomonas aeruginosa* activates caspase 1 through IPAF. Proc Natl Acad Sci USA. 2008;105(7):2562–2567. doi: 10.1073/pnas.0712183105 18256184 PMC2268176

[81] Sen-Kilic E, Huckaby AB, Damron FH, et al. *P. aeruginosa* type III and type VI secretion systems modulate early response gene expression in type II pneumocytes *in vitro*. BMC Genomics. 2022;23(1):345. doi: 10.1186/s12864-022-08554-0 35508983 PMC9068226

[82] Kolbe U, Yi B, Poth T, et al. Early cytokine induction upon *Pseudomonas aeruginosa* infection in murine precision cut lung slices depends on sensing of bacterial viability. Front Immunol. 2020;11:598636. doi: 10.3389/fimmu.2020.598636 33250899 PMC7673395

[83] Bomberger JM, Maceachran DP, Coutermarsh BA, et al. Long-distance delivery of bacterial virulence factors by *Pseudomonas aeruginosa* outer membrane vesicles. PLOS Pathog. 2009;5(4):e1000382. doi: 10.1371/journal.ppat.1000382 19360133 PMC2661024

[84] Qin S, Xiao W, Zhou C, et al. *Pseudomonas aeruginosa*: pathogenesis, virulence factors, antibiotic resistance, interaction with host, technology advances and emerging therapeutics. Signal Transduct Target Ther. 2022;7(1):199. doi: 10.1038/s41392-022-01056-1 35752612 PMC9233671

[85] Kim SI, Kim S, Kim E, et al. Secretion of Salmonella pathogenicity island 1-encoded type III secretion system effectors by outer membrane vesicles in Salmonella enterica serovar Typhimurium. Front Microbiol. 2018;9:2810. doi: 10.3389/fmicb.2018.02810 30532744 PMC6266720

[86] Bai J, Kim SI, Ryu S, et al. Identification and characterization of outer membrane vesicle-associated proteins in Salmonella enterica serovar Typhimurium. Infect Immun. 2014;82(10):4001–4010. doi: 10.1128/IAI.01416-13 24935973 PMC4187864

[87] Sirisaengtaksin N, O’Donoghue EJ, Jabbari S, et al. Bacterial outer membrane vesicles provide an alternative pathway for trafficking of Escherichia coli O157 type III secreted effectors to epithelial cells. mSphere. 2023;8(6):e0052023. doi: 10.1128/msphere.00520-23 37929984 PMC10732017

[88] Armstrong DA, Lee MK, Hazlett HF, et al. Extracellular vesicles from *Pseudomonas aeruginosa* suppress MHC-related molecules in human lung macrophages. ImmunoHorizons. 2020;4(8):508–519. doi: 10.4049/immunohorizons.2000026 32819967 PMC7728166

[89] Wauters M, Bollé L, Van Den Bossche S, et al. Identification of Dksa as a novel pro-inflammatory mediator of *Pseudomonas aeruginosa* under conditions mimicking chronic cystic fibrosis lung infection. 2025. 10.1080/21505594.2026.2670050 PMC13170382 42117785

[90] Wauters M, Crabbe A. Identification of Dksa as a novel pro-inflammatory mediator of *Pseudomonas aeruginosa* under conditions mimicking chronic cystic fibrosis lung infection. Zenodo. 2025. doi:10.5281/zenodo.19369593. PMC13170382 42117785

