# Supplementary material for: Identification of DksA as a novel pro-inflammatory mediator of Pseudomonas aeruginosa under conditions mimicking chronic cystic fibrosis lung infection
Source: Virulence. 2026 May 12;17(1):2670050. doi: 10.1080/21505594.2026.2670050 (PMC13170382; doi:10.1080/21505594.2026.2670050)
Supplement: Manuscript_DksA_Virulence_Resubmission_Supplem.docx [file KVIR_A_2670050_SM6044.docx]

# SUPPLEMENTARY DATA:

# Identification of DksA as a novel pro-inflammatory mediator of *Pseudomonas aeruginosa* under conditions mimicking chronic cystic fibrosis lung infection

#### Authors

#### Merel Wauters (0000-0003-4715-9759) ^1^, Laura Bollé ^1,2,3^ (0000-0002-2769-1943), Gilles De Meester ^1^ (0000-0002-9855-9843), Sara Van den Bossche (0000-0001-7839-8758) ^1^, Lucia Grassi (0000-0001-8607-9298) ^1^, Delphi Van Haver (0000-0002-4659-5631) ^4,5,6^, Sara Dufour (0000-0003-2036-7849) ^4,5,6^, Simon Devos (0000-0002-0623-433X) ^4,5,6^, Francis Impens (0000-0003-2886-9616) ^5,6^, Eva Van Braeckel (0000-0002-7242-0747) ^2,3^, Anna K.H. Hirsch (0000-0001-8734-4663) ^7,8,9^, Marvin Whiteley (0000-0002-4933-9983) ^10^, Xavier Saelens (0000-0002-3861-6965) ^5,11^ and Aurélie Crabbé (0000-0003-3084-4418) ^1^

#### Affiliations

#### ^1^ Laboratory of Pharmaceutical Microbiology, Ghent University, Ghent, Belgium

^2^ Respiratory Infection and Defense Lab (RIDL), Department of Internal Medicine and Paediatrics, Faculty of Medicine and Health Sciences, Ghent University, Ghent, Belgium

^3^ Department of Respiratory Medicine, Ghent University Hospital, Ghent, Belgium

#### ^4^ VIB Proteomics Core, VIB, Ghent, Belgium

#### ^5^ Center for Medical Biotechnology, VIB, Ghent, Belgium

#### ^6^ Department of Biomolecular Medicine, Ghent University, Ghent, Belgium

^7^ Helmholtz Institute for Pharmaceutical Research Saarland (HIPS) – Helmholtz Centre for Infection Research (HZI), Campus E8.1, 66123, Saarbrücken, Germany

^8^ Saarland University, Department of Pharmacy, Campus E8.1, 66123, Saarbrücken, Germany

^9^ PharmaScienceHub, Campus A 2.3, 66123 Saarbrücken, Germany

^10^ School of Biological Sciences and Center for Microbial Dynamics and Infection, Georgia Institute of Technology, Atlanta, Georgia, USA

#### ^11^ Department of Biochemistry and Microbiology, Ghent University, Ghent, Belgium

#### Keywords

*Pseudomonas aeruginosa*, virulence factors, inflammation, cystic fibrosis, proteomics, transcriptomics

#### Corresponding author

Aurélie Crabbé (aurelie.crabbe@ugent.be)

The data presented in ***Figure S.1*** was reproduced from our previous work; no new experiments were conducted (1).


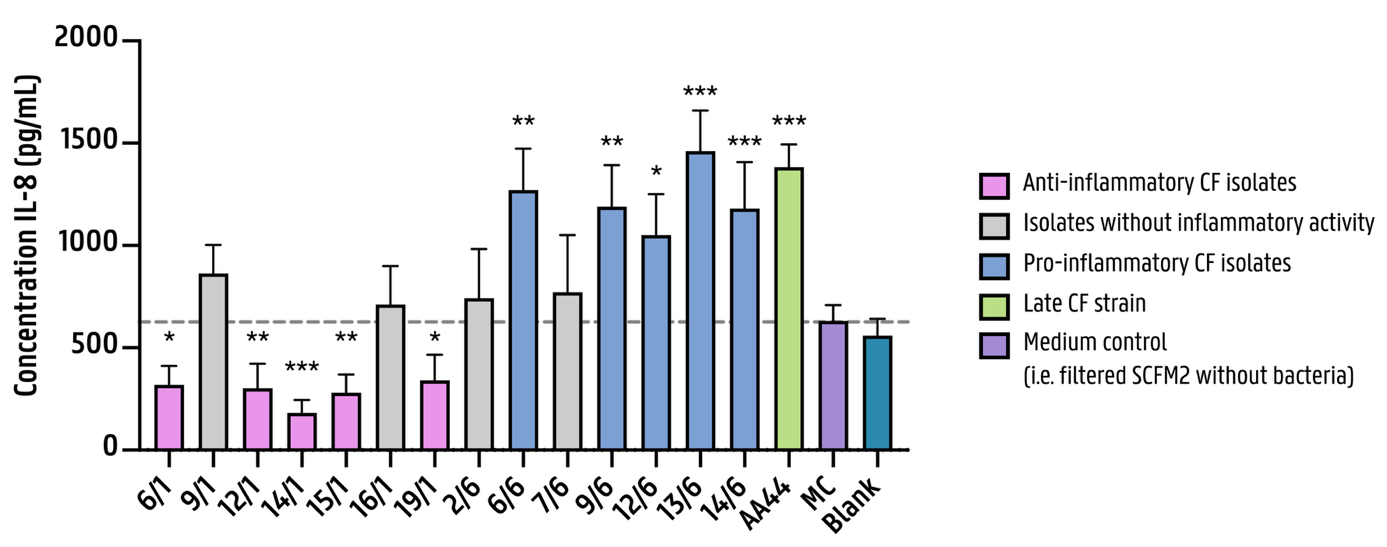


***Figure S.1: A diverse inflammatory response was observed between isolates, ranging from undetectable to significant levels of IL-8 secretion by 3D lung epithelial cells:*** *Cytokine release was quantified in organotypic 3D lung aggregates following exposure to 40% (v/v) P. aeruginosa cell-free supernatants for 4 h. SCFM2 medium alone served as the medium control (MC), while the blank represents untreated A549 cells cultured in GTSF-2 medium without FBS and antibiotics. Both blank and MC indicate the basal level of IL-8 produced in the absence of a pro-inflammatory stimulus. The positive control was 10% (v/v) supernatant of P. aeruginosa AA44 grown in SCFM2. IL-8 levels were determined using ELISA with cytokine concentration displayed on the y-axis. (n=3–7, statistical analysis using a Tweedie generalized linear mixed model was conducted on the absolute data, which was then compared to the MC baseline (horizontal dotted line indicating the MC mean). * p < 0.05, ** p < 0.01, *** p < 0.001, error bars represent the standard error). Data previously published in* (1)*.*


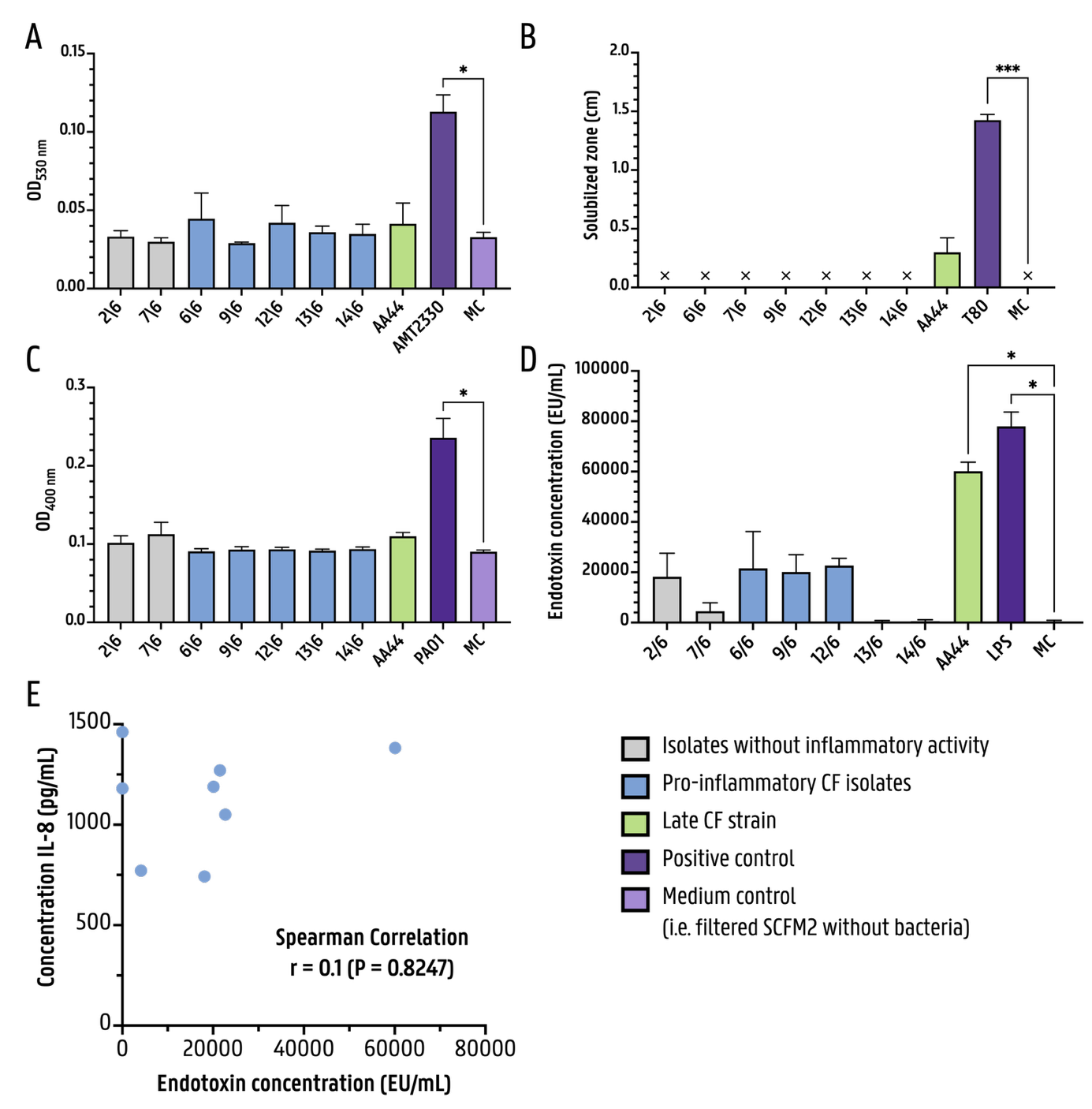


***Figure S.2: Virulence factors quantified in P. aeruginosa cell-free supernatants or bacterial suspension*** *(5 x 10^7^ CFU/mL for rhamnolipid assay): (A) Pyocyanin was quantified using chloroform-HCl extraction. Strain AMT 0023-30 served as a positive control. (B) Rhamnolipids were assessed by plating bacterial suspensions on CTAB-MB plates for 48 h under microaerophilic conditions. The size of the halos minus the diameter of the cut-out holes indicated rhamnolipid production. Tween-80 (100 mM) was used as a positive control. An ‘X’ on the x-axis indicates that no measurable halo was present. (C) Pyoverdine levels were measured via absorbance at 400 nm. Strain PAO1 served as a positive control. (D) Endotoxin levels were quantified using the Pierce Chromogenic Endotoxin Quant Kit. LPS from P. aeruginosa serotype 10 (1 mg/mL) was used as a positive control. Although CF isolates 6/6, 9/6, and 12/6 reached endotoxin levels of ~20,000 EU/mL, these levels were not significantly different from the SCFM2 control (i.e., p = 0.193, 0.176, 0.141, respectively). (E) Scatter plot illustrating the relationship between endotoxin levels in the cell-free supernatants of clinical CF isolates (x-axis), and IL-8 release from 3D lung epithelial aggregates (y-axis) following exposure to 40% (v/v) of the corresponding cell-free supernatants for 4 h. (For assays A-D: n ≥ 3, statistical analysis was performed using the non-parametric Kruskal–Wallis ANOVA comparing to the MC, followed by the Benjamini–Hochberg correction (FDR of 5%). For E: n = 8, statistical analysis was performed using a two-tailed Spearman correlation, * p < 0.05, ** p < 0.01, *** p < 0.001, error bars represent standard error)*

*
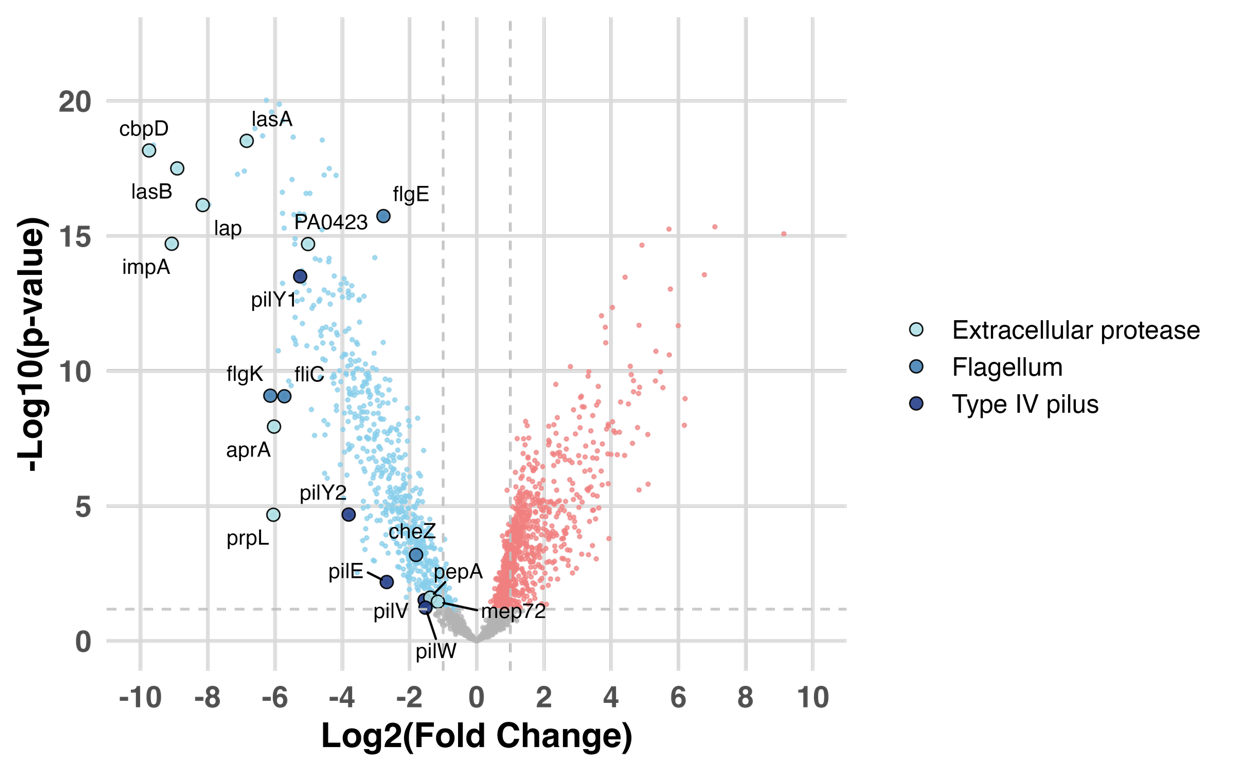
*

***Figure S.3: Extracellular proteases and proteins associated with the flagellum or type IV pilus are downregulated in the pro-inflammatory P. aeruginosa isolate group****: A volcano plot was generated to display the differences in protein abundance between the pro- and immunosuppressive isolate group. The difference is represented as log_2_(Fold Change) on the x-axis, plotted against -log_10_(p-values) on the y-axis, derived from a two-sample t-test. Symbols corresponding to extracellular proteases, and proteins associated with the flagellum or type IV pilus are enlarged and labeled. The horizontal dashed line indicates the significance threshold, determined using a permutation-based FDR correction of 5%, and the vertical dashed line marks a log_2_(Fold Change) = |1|.*

*
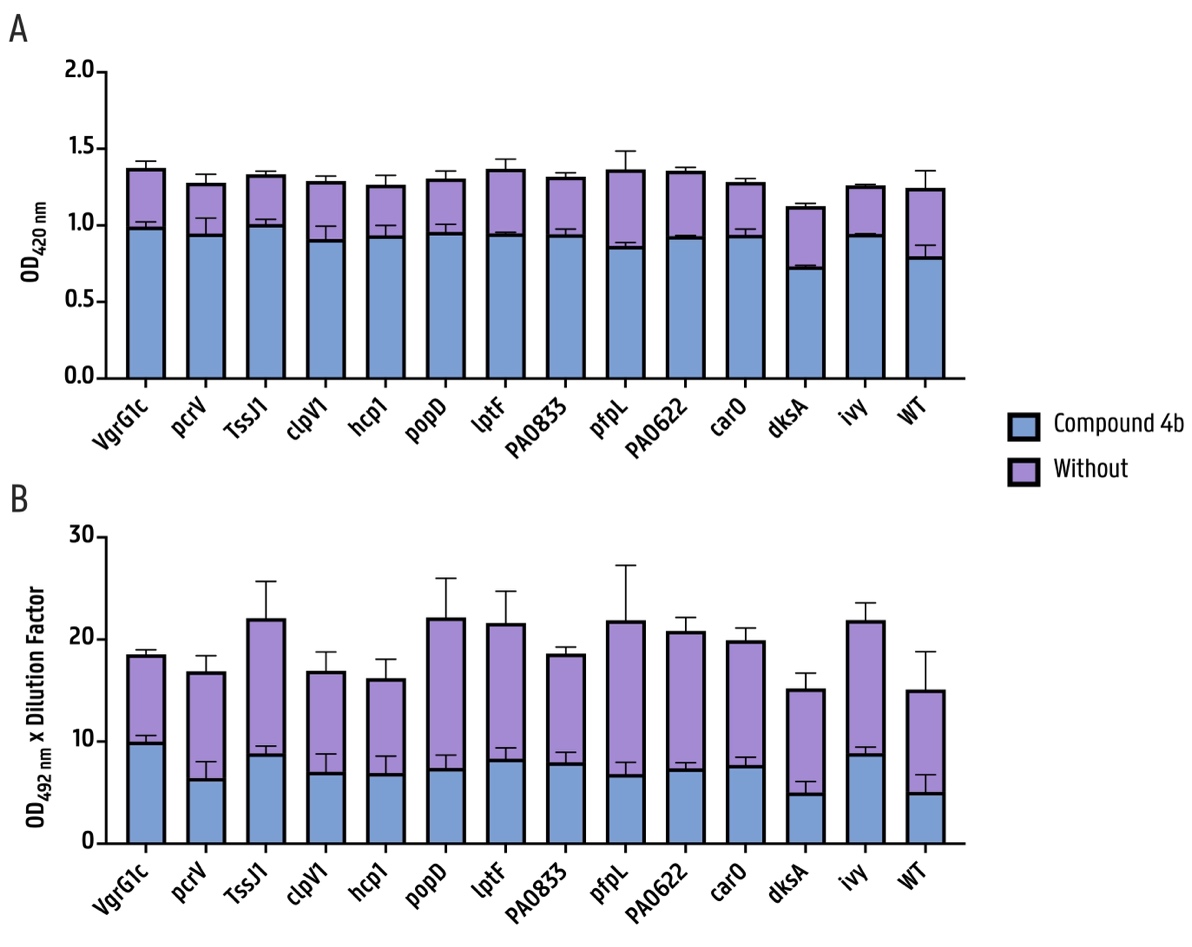
*

***Figure S.4: Proteolytic activity in the cell-free culture supernatant of P. aeruginosa PA14 Tn mutant strains:*** *(A) Proteolytic activity of the cell-free supernatants (40% (v/v)) from the PA14 Tn mutant strains was determined by the azocasein colorimetric assay, in the presence or absence of 50 μM LasB inhibitor* ***4b*** *(0.5% (v/v)). (B) Elastolytic activity of the cell-free supernatants (40% (v/v)) from the PA14 Tn mutant strains was determined by the Elastin-Congo red assay, in the presence or absence of 50 μM compound* ***4b*** *(0.5% (v/v)). High absorbance values correspond with high proteolytic/elastolytic activity. (For all assays: n ≥ 3, statistical analysis was performed using a mixed-effects model with REML estimation, error bars represent the standard error).*

***
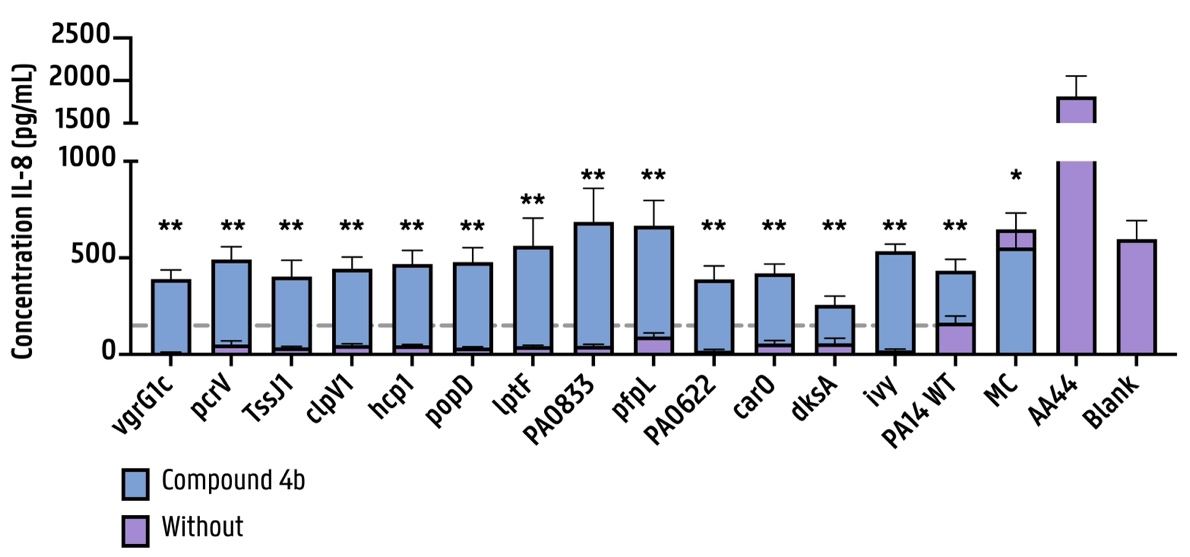
***

***Figure S.5: IL-8 produced by 3D lung aggregates following exposure to cell-free supernatants from P. aeruginosa PA14 Tn mutant strains:*** *IL-8 release was measured in an organotypic 3D lung cell culture model after 4 h exposure to 40% (v/v) P. aeruginosa cell-free supernatants, in the presence or absence of 50 μM LasB inhibitor* ***4b*** *(0.5% (v/v)). Differences in IL-8 release in the presence or absence of compound* ***4b*** *were assessed for each Tn mutant. SCFM2 alone served as the medium control (MC), while the blank represents untreated A549 cells cultured in GTSF-2 medium without FBS. Both controls reflect basal cytokine levels in the absence of a pro-inflammatory stimulus. (n ≥ 3, statistical analysis was performed using multiple Mann-Whitney U tests followed by the Benjamini-Hochberg correction (FDR = 5%) for multiple testing to assess the effect of the inhibitor for each pair (asterisk above bars indicate significant differences). Additionally, the non-parametric Kruskal–Wallis ANOVA followed by Benjamini–Hochberg correction (5% FDR) was used to compare IL-8 release (in the absence of inhibitor* ***4b****) induced by each Tn mutant with that of the PA14 WT supernatant (with horizontal dotted line indicating the group mean). No significant differences were observed (not shown), * p < 0.05, ** p < 0.01, *** p < 0.001, error bars represent the standard error).*

*
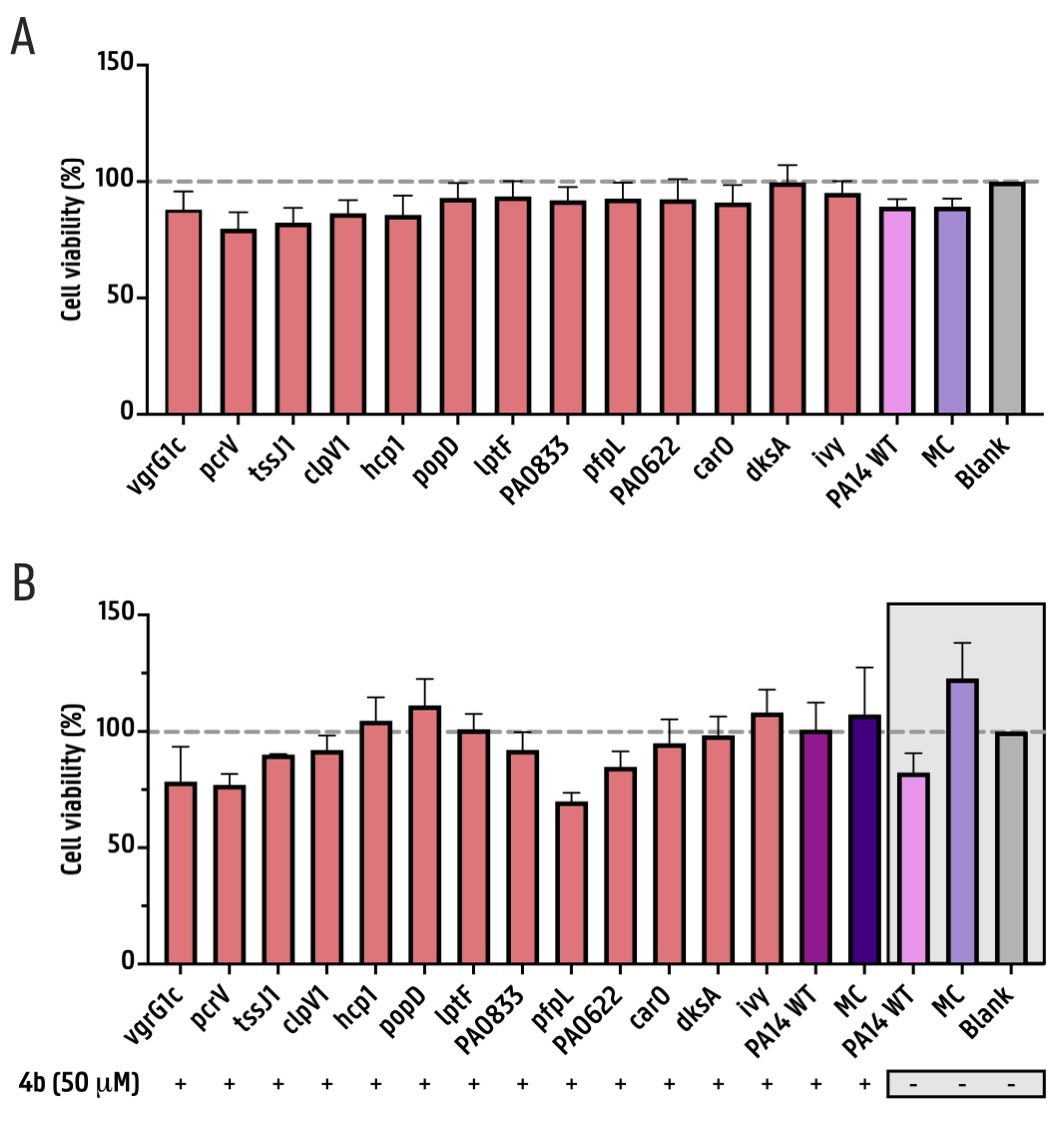
*

***Figure S.6: Cytotoxic response of 3D lung aggregates to cell-free supernatants from PA14 Tn mutant strains, with or without LasB inhibitor 4b:*** *(A) Cell-viability of the organotypic 3D lung cell culture model following 4h exposure to 40% (v/v) P. aeruginosa cell-free supernatants. (B) Cell-viability of the organotypic 3D lung cell culture model after 4h exposure to 40% (v/v) P. aeruginosa cell-free supernatants, in the presence or absence of 50 μM LasB inhibitor* ***4b*** *(0.5% (v/v)). Viability was determined by LDH release using an intracellular LDH assay. The blank control represents untreated A549 cells in GTSF-2 medium without FBS, while SCFM2 alone serves as the medium control (MC). Cell-viability (y-axis) is expressed relative to the blank, with the horizontal dotted line indicating 100% viability. (For all assays: n ≥ 3, statistical analysis of the relative data was conducted using the non-parametric Kruskal–Wallis ANOVA as described in (2), multiple testing correction was performed using the Benjamini–Hochberg procedure with a 5% FDR, no significant differences were observed (not shown), * p < 0.05, error bars represent the standard error).*


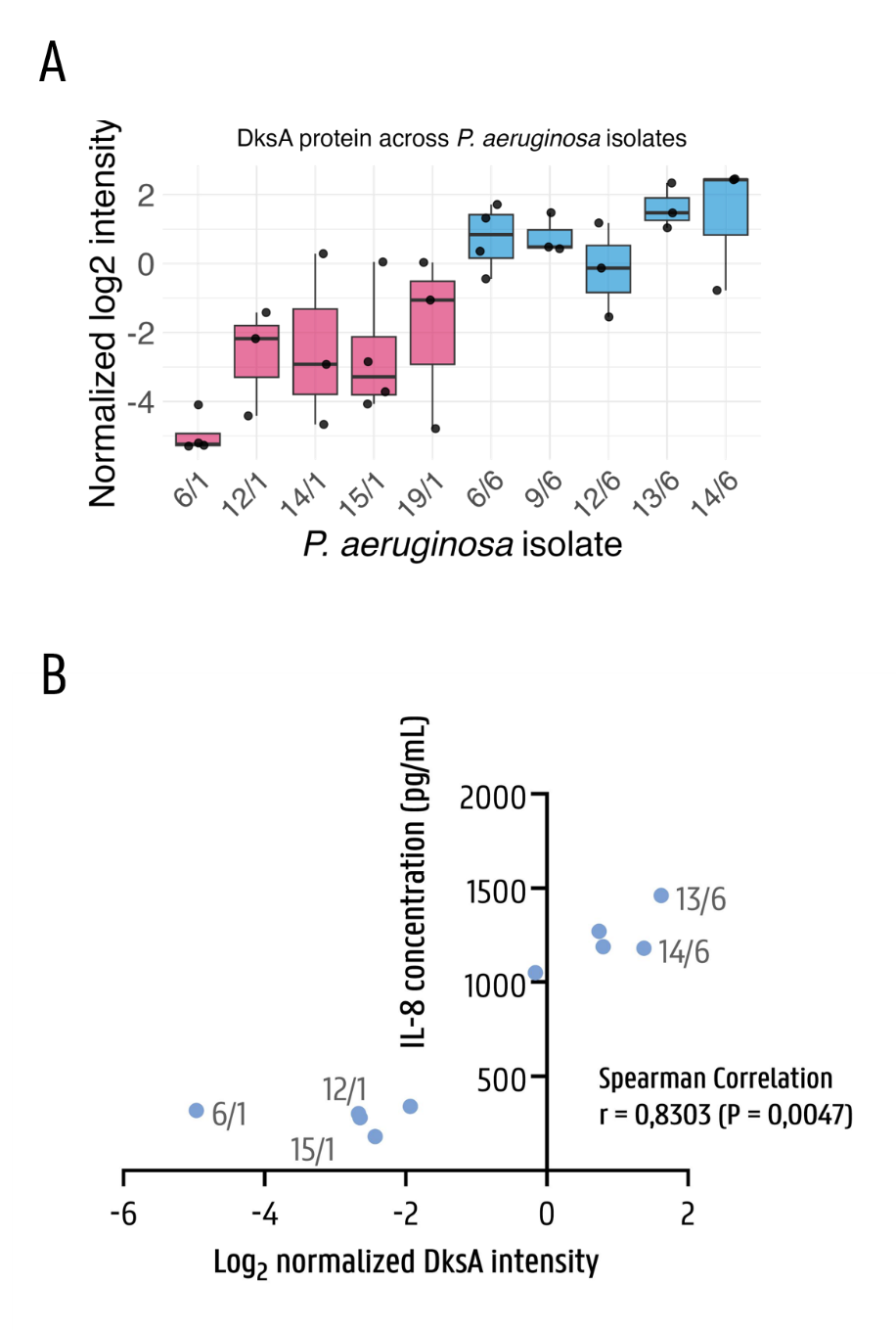


***Figure S.7: DksA abundance correlates with IL-8 induction****. (A) Boxplot showing the abundance of DksA across the samples. Immunosuppressive isolates are represented in pink, while isolates with pro-inflammatory activity are shown in blue. (B) Scatter plot illustrating the relationship between DksA abundance in the cell-free supernatants of clinical CF isolates (x-axis), and IL-8 release from 3D lung epithelial aggregates (y-axis) following exposure to 40% (v/v) of the corresponding cell-free supernatants for 4 h. (For B: n = 10, statistical analysis was performed using a two-tailed Spearman correlation).*


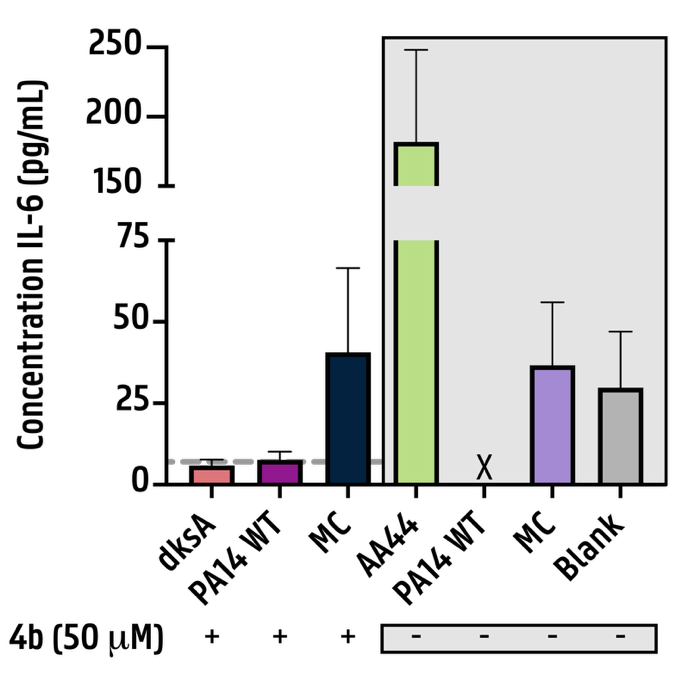


***Figure S.8: IL-6 produced by 3D lung aggregates following exposure to cell-free supernatants from P. aeruginosa PA14 dksA::Tn mutant strain.*** *IL-6 release was measured in an organotypic 3D lung cell culture model after 4 h exposure to 40% (v/v) P. aeruginosa cell-free supernatants, in the presence or absence of 50 μM LasB inhibitor* ***4b*** *(0.5% (v/v)). SCFM2 alone served as the medium control (MC), while the blank represents untreated A549 cells cultured in GTSF-2 medium without FBS. Both controls reflect basal cytokine levels in the absence of a pro-inflammatory stimulus. (n = 3, statistical analysis was performed using a Mann-Whitney U test to compare IL-6 release induced by the dksA::Tn mutant treated with inhibitor to that induced by the PA14 WT supernatant treated with inhibitor. The horizontal dotted line indicates the group mean of PA14 WT supernatant with inhibitor. No significant differences were observed (not shown), error bars represent the standard error).*

*
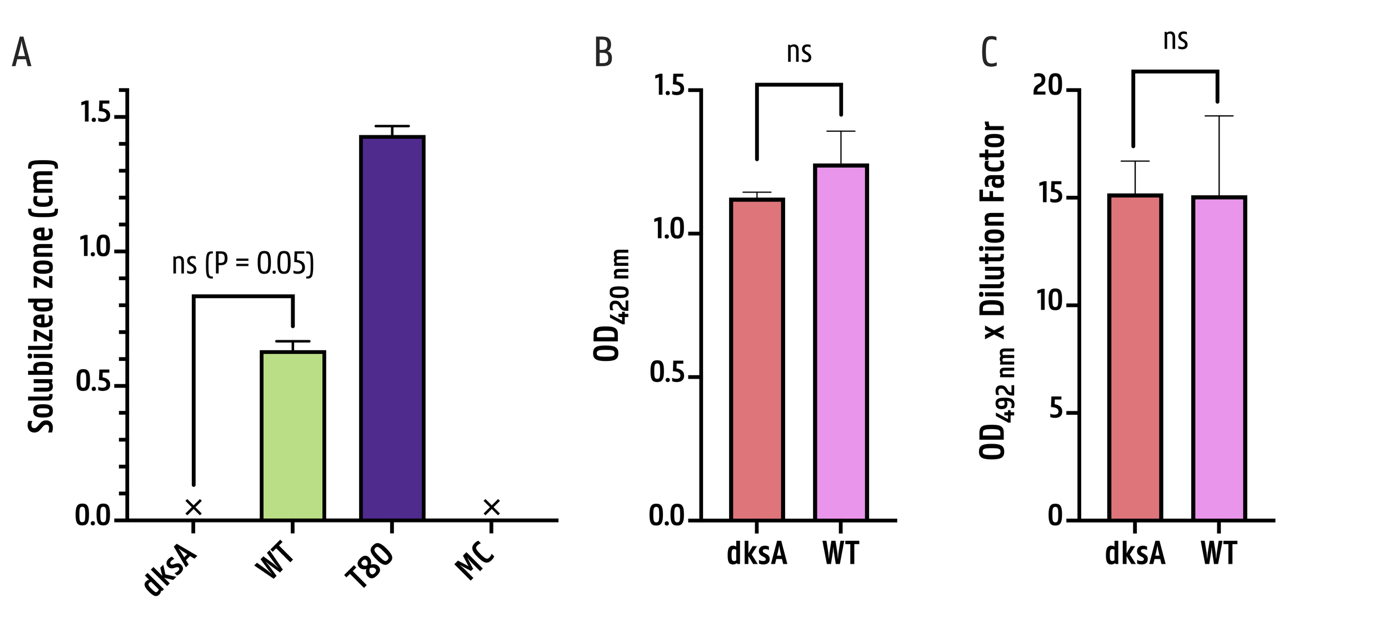
*

***Figure S.9: Virulence factors were quantified in PA14 WT and PA14 dksA::Tn cell-free supernatant or bacterial suspension of PA14 WT and PA14 dksA::Tn*** *(5 x 10^7^ CFU/mL for rhamnolipid assay): (A) Rhamnolipids were assessed by plating bacterial suspensions on CTAB-MB plates for 48 h under microaerophilic conditions. The size of the halos minus the diameter of the cut-out wells indicated rhamnolipid production. Tween-80 was used as a positive control. An ‘X’ on the x-axis indicates that no measurable halo was present. (B) Proteolytic activity of PA14 WT and PA14 dksA::Tn cell-free supernatant was determined by the azocasein colorimetric assay. (C) Elastolytic activity of PA14 WT and PA14 dksA::Tn cell-free supernatant was determined by the Elastin-Congo red assay. (For all assays: n = 3, statistical analysis was performed using a one-tailed non-parametric Mann–Whitney U test comparing PA14 dksA::Tn to PA14 WT, * p < 0.05, error bars represent standard error).*

***
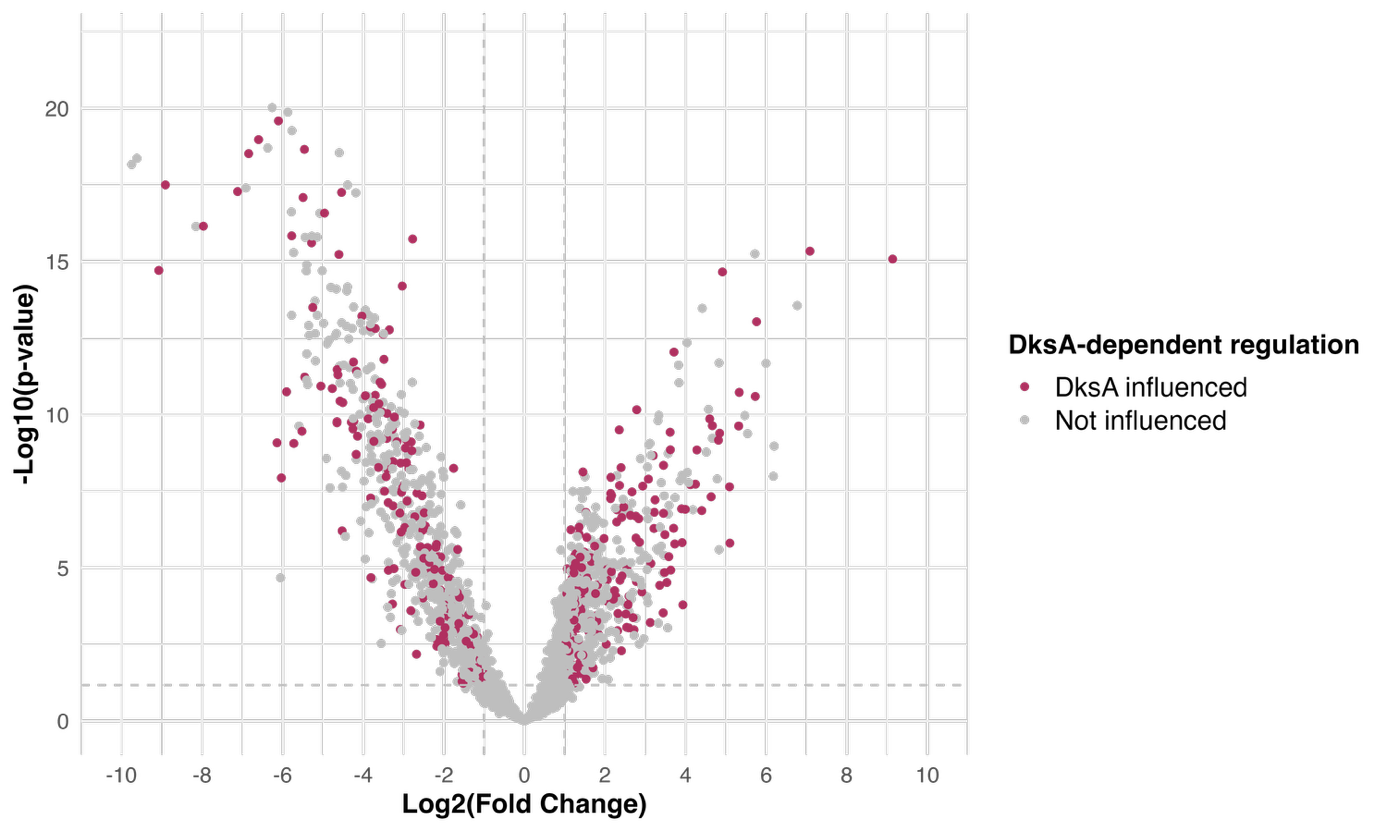
***

***Figure S.10: dksA-dependent regulation of differentially expressed proteins in pro- versus immunosuppressive isolates:*** *Volcano plot showing protein abundance differences between pro-inflammatory and immunosuppressive isolates. Differences are represented as log_2_(Fold Change) on the x-axis, against -log_10_(p-values) on the y-axis, calculated using a two-sample t-test. Proteins significantly differentially expressed with a fold change > |2| under dksA regulation, based on data from Fortuna et al. (3), are highlighted. The horizontal dashed line indicates the significance threshold (5% permutation-based FDR), and the vertical dashed line marks a log_2_(Fold Change) = |1|.*

*
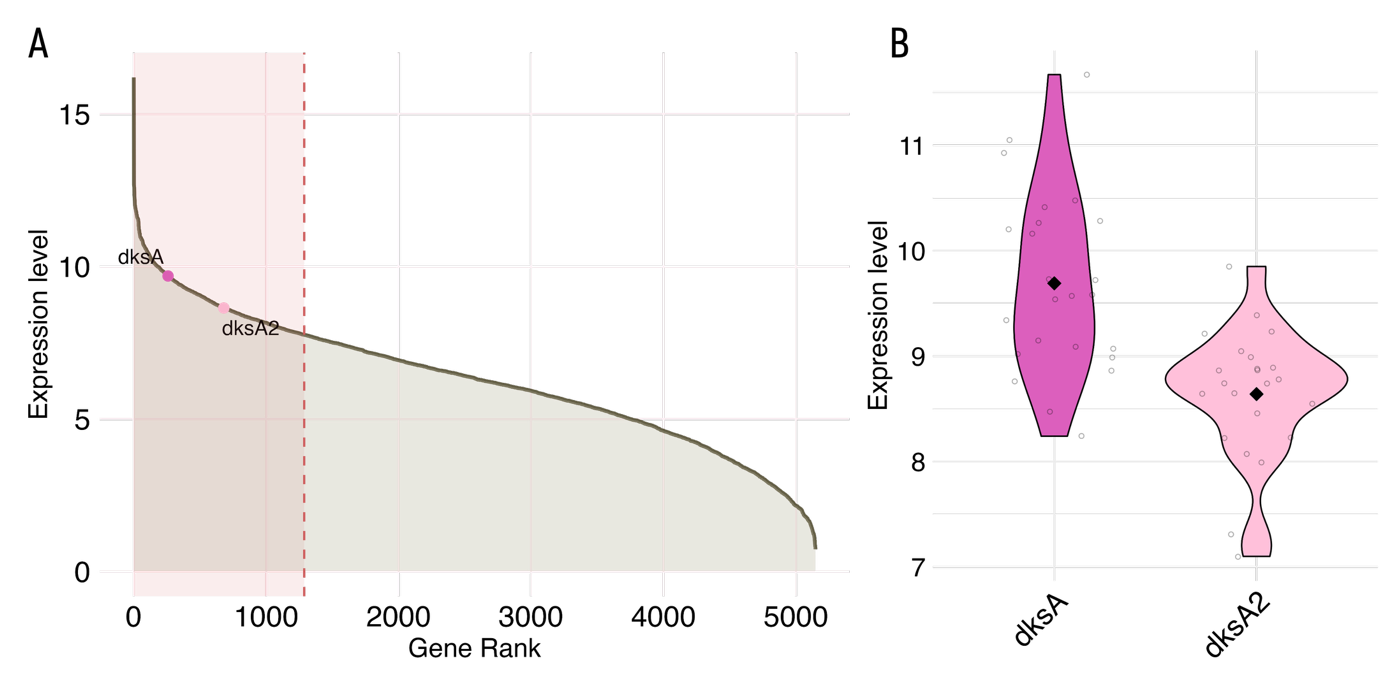
*

***Figure S.11:*** ***Expression profile of dksA and dksA2 across 24 P. aeruginosa transcriptomes derived from CF sputum samples (data from Lewin et al. (4)):*** *(A) Cumulative gene expression plot displaying genes ranked by their average expression (VST-normalized count data) across the 24 CF sputum-derived P. aeruginosa transcriptomes. dksA and dksA2 are labeled and the top quartile (25%) of most highly expressed genes is highlighted with a transparent red box; (B) Violin plots depicting the distribution and variability of expression levels for dksA and dksA2 across all 24 samples with each violin representing one gene.*

*
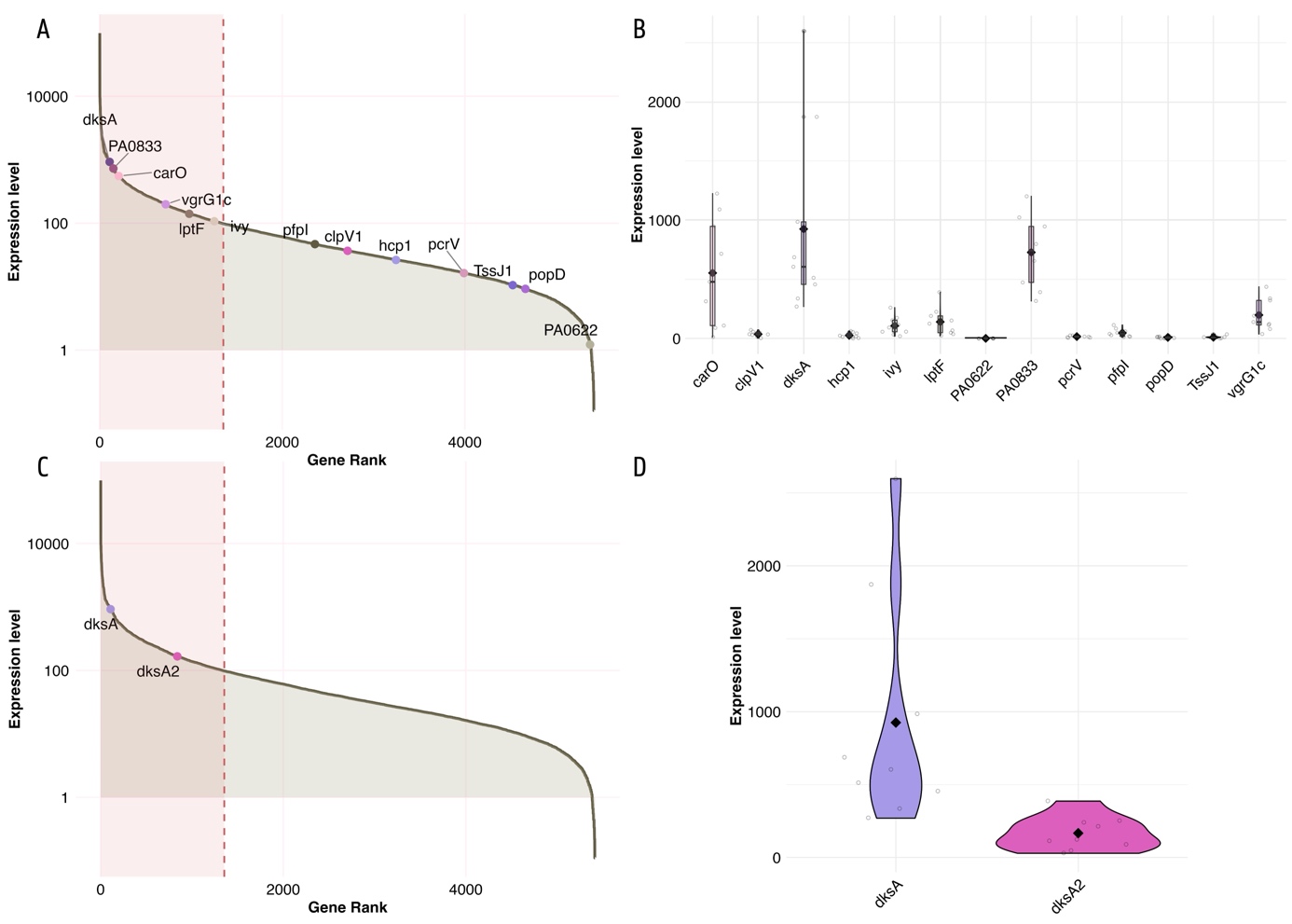
*

***Figure S.12:*** ***Expression profile of mediators of interest across nine P. aeruginosa transcriptomes derived from CF sputum samples belonging to four different pwCF chronically infected with P. aeruginosa:*** *data were obtained from the P. aeruginosa database (originally derived from Rossi et al.* *(5))* *(A) Cumulative gene expression plot displaying genes ranked by their average expression (normalized count data) across the nine CF sputum-derived P. aeruginosa transcriptomes. Proteins of interest are labeled, and the top quartile (25%) of most highly expressed genes is highlighted with a transparent red box; (B) Violin plots depicting the distribution and variability of expression levels for selected mediators across all nine samples with each violin representing one gene. (C) Cumulative gene expression plot with dksA and dksA2 labeled. (D) Violin plots depicting the distribution and variability of expression levels for dksA and dksA2 across all nine samples.*

***Table S.1: Sixty-two virulence-associated proteins were upregulated in isolates that triggered pro-inflammatory activity in 3D lung aggregates (p < 0.05, log_2_(Fold Change) > 1).*** *The complete list of proteins and their DAVID functional annotations is provided on Zenodo.*

| **Gene names** | **Protein name** | **PA number** | **UniProt ID** |
| --- | --- | --- | --- |
| alg44 | alginate biosynthesis protein Alg44 | PA3542 | Q9HY69 |
| algX | alginate biosynthesis protein AlgX | PA3546 | Q51372 |
| algL | alginate lyase | PA3547 | Q06749 |
| algF | alginate o-acetyltransferase AlgF | PA3550 | Q06062 |
| mucA | sigma factor AlgU negative regulator MucA | PA0763 | P38107 |
| algD | GDP-mannose 6-dehydrogenase AlgD | PA3540 | P11759 |
| algU | RNA polymerase sigma factor AlgU | PA0762 | Q06198 |
| tse6 | NAD(P)(+) glycohydrolase toxin Tse6 | PA0093 | Q9I739 |
| vgrG1c | Type VI secretion system spike protein VgrG1c | PA2685 | Q9I0F3 |
| PA4624 | cyclic diguanylate-regulated TPS partner B, CdrB | PA4624 | Q9HVG7 |
| sctF | type III export protein PscF | PA1719 | P95434 |
| pcrV | type III secretion protein PcrV | PA1706 | G3XD49 |
| PA0078 | hypothetical protein | PA0078 | Q9I754 |
| tssK1 | hypothetical protein | PA0079 | Q9I753 |
| PA0080 | hypothetical protein | PA0080 | Q9I752 |
| tssA1 | hypothetical protein | PA0082 | Q9I750 |
| tssC1 | hypothetical protein | PA0084 | Q9I748 |
| secD | preprotein translocase subunit SecD | PA3821 | Q9HXI1 |
| secY | preprotein translocase subunit SecY | PA4243 | Q9HWF5 |
| clpV1 | secretion protein ClpV1 | PA0090 | Q9I742 |
| ftsY | signal recognition particle receptor FtsY | PA0373 | Q9I6C1 |
| tatA | twin-arginine translocation protein TatA | PA5068 | Q9HUB5 |
| tssM1 | type VI secretion protein IcmF | PA0077 | Q9I755 |
| hcp1 | protein secretion apparatus assembly protein | PA0085 | Q9I747 |
| PA2633 | hypothetical protein | PA2633 | Q9I0K5 |
| popD | translocator outer membrane protein PopD | PA1709 | Q9I323 |
| fliN | flagellar motor switch protein FliN | PA1444 | Q51466 |
| waaL | O-antigen ligase WaaL | PA4999 | Q9HUG6 |
| wbpI | UDP-2,3-diacetamido-2,3-dideoxy-D-glucuronate 2-epimeras | PA3148 | G3XD61 |
| lpxC | UDP-3-O-[3-hydroxymyristoyl] N-acetylglucosamine deacetylase | PA4406 | P47205 |
| pslB | biofilm formation protein PslB | PA2232 | Q9I1N7 |
| rmlA | glucose-1-phosphate thymidylyltransferase | PA5163 | Q9HU22 |
| waaF | heptosyltransferase II | PA5012 | G3XD35 |
| PA3828 | Lipopolysaccharide export system permease protein | PA3828 | Q9HXH4 |
| PA4069 | hypothetical protein | PA4069 | Q9HWV9 |
| lptH | hypothetical protein | PA4460 | Q9HVV7 |
| PA5455 | hypothetical protein | PA5455 | Q9HTB5 |
| pagL | lipid A 3-O-deacylase | PA4661 | Q9HVD1 |
| PA0011 | lipid A biosynthesis lauroyl acyltransferase | PA0011 | Q9I7B5 |
| wbpM | nucleotide sugar epimerase/dehydratase WbpM | PA3141 | Q9HZ86 |
| PA4735 | hypothetical protein | PA4735 | Q9HV64 |
| galU | UTP-glucose-1-phosphate uridylyltransferase | PA2023 | Q9I291 |
| PA0729 | Pf4 inhibition toxin, PfiT | PA0729 | Q9I5J9 |
| lptF | Lipotoxin F | PA3692 | Q9HXU8 |
| PA0833 | hypothetical protein | PA0833 | Q9I5A7 |
| clpP1 | ATP-dependent Clp protease proteolytic subunit | PA1801 | Q9I2U1 |
| pfpI | protease PfpI | PA0355 | Q9I6D8 |
| eco | ecotin | PA2755 | Q9I088 |
| PA1832 | protease | PA1832 | Q9I2R3 |
| prc | tail-specific protease | PA3257 | Q9HYY3 |
| PA3649 | zinc metalloprotease | PA3649 | Q9HXY3 |
| lepB | signal peptidase I | PA0768 | Q9I5G7 |
| pvdH | diaminobutyrate--2-oxoglutarate aminotransferase | PA2413 | Q9I168 |
| fpvA | ferripyoverdine receptor | PA2398 | P48632 |
| piuA | iron transport outer membrane receptor | PA4514 | G3XCY8 |
| amrZ | alginate and motility regulator Z | PA3385 | G3XCY4 |
| algR | alginate biosynthesis regulatory protein AlgR | PA5261 | P26275 |
| pilO | type 4 fimbrial biogenesis protein PilO | PA5042 | G3XD51 |
| pilG | pilus biosynthesis/twitching motility protein PilG | PA0408 | P46384 |
| pilH | twitching motility protein PilH | PA0409 | P43501 |
| PA0622 | bacteriophage protein | PA0622 | G3XD39 |
| typA | regulatory protein TypA | PA5117 | Q9HU67 |

***Table S.2:*** *Upregulated differentially expressed proteins in the pro-inflammatory isolates group. In total, 605 proteins were upregulated. Differential expression analysis between the pro-inflammatory and immunosuppressive isolate group was assessed using a two-sample t-test with multiple testing correction applied via a permutation-based FDR of 5%. Log_2_(Fold Change) values are reported.*

***Table S.3:*** *Downregulated differentially expressed proteins in the pro-inflammatory isolates group. In total, 631 proteins were downregulated. Multiple extracellular proteases, as well as proteins associated with Type IV pilus and flagellum-dependent motility (See Figure S.3) are highlighted in light blue. Differential expression analysis between the pro-inflammatory and immunosuppressive isolate group was assessed using a two-sample t-test with multiple testing correction applied via a permutation-based FDR of 5%. Log_2_(Fold Change) values are reported.*

***Table S.4: dksA-regulated proteins differentially expressed (p < 0.05, log_2_(Fold Change) > |1|) between pro-inflammatory and immunosuppressive isolates.*** *DksA regulon based on data from Fortuna et al. (3). Differential expression analysis between the pro-inflammatory and immunosuppressive isolate group was assessed using a two-sample t-test with multiple testing correction applied via a permutation-based FDR of 5%.* *Log_2_(Fold Change) values are reported.*

***Table S.2****,* ***Table S.3****, and* ***Table S.4 (xlsx)*** *are provided on Zenodo.*

# References

1. Wauters M, Van Den Bossche S, Grassi L*, et al.* Unraveling the Immunosuppressive Role of Elastase B Produced by Cystic Fibrosis Isolates of *Pseudomonas Aeruginosa* in an Organotypic 3d Lung Epithelial Cell Model. Virulence. 2025;16(1).

2. Van den Bossche S, Vandeplassche E, Ostyn L*, et al.* Bacterial Interference with Lactate Dehydrogenase Assay Leads to an Underestimation of Cytotoxicity. Front Cell Infect Microbiol. 2020;10:494.

3. Fortuna A, Bähre H, Visca P*, et al.* The Two *Pseudomonas Aeruginosa* Dksa Stringent Response Proteins Are Largely Interchangeable at the Whole Transcriptome Level and in the Control of Virulence-Related Traits. Environmental Microbiology. 2021;23(9):5487–504.

4. Lewin GR, Kapur A, Cornforth DM*, et al.* Application of a Quantitative Framework to Improve the Accuracy of a Bacterial Infection Model. Proceedings of the National Academy of Sciences. 2023;120(19):e2221542120.

5. Rossi E, Falcone M, Molin S*, et al.* High-Resolution in Situ Transcriptomics of *Pseudomonas Aeruginosa* Unveils Genotype Independent Patho-Phenotypes in Cystic Fibrosis Lungs. Nat Commun. 2018;9(1):3459.
